# Mineral-Enriched Biochars from Co-Pyrolysis of Sewage Sludge, Sawdust, and Basaltic Rocks: Soil Amendment Potential and Carbon Stability

**DOI:** 10.1021/acsomega.6c07681

**Published:** 2026-10-19

**Authors:** Maíra Lopes D̀Ávila, José Ferreira Lustosa Filho, Éder de Souza Martins, Giuliano Marchi, Cícero Célio de Figueiredo

**Affiliations:** † Faculdade de Agronomia e Medicina Veterinária, 28127 Universidade de Brasília, 70910-970, Brasília, Federal District, Brazil; ‡ Empresa Brasileira de Pesquisa Agropecuária, Embrapa Cerrados, 73310-970, Planaltina, Federal District, Brazil

## Abstract

Co-pyrolysis of sewage sludge (SS) and sawdust (SD) with basaltic minerals may produce nutrient-enriched biochars for soil amendment and long-term carbon (C) persistence. However, the effects of mineral incorporation on C transformations and the interpretation of conventional stability indices remain poorly understood. To address this gap, we compared SS and SD copyrolyzed with zeolitic basalt (ZB) and metabasalt (MB). The resulting materials, produced at 500 °C, were characterized by proximate and elemental analyses, X-ray diffraction, energy-dispersive X-ray spectroscopy, Fourier-transform infrared spectroscopy, thermogravimetric analysis, and nutrient and carbon-stability assessments. Mineral addition increased biochar yields to 70–73%, whereas SD-derived biochar exhibited the highest fixed C and thermostable fractions. Materials obtained by co-pyrolyzing SD with ZB or MB, and SS with ZB, showed high thermal resistance. Mineral-enriched biochars had higher ash contents and contained silicate-rich phases, modifying their mineralogical composition and nutrient profiles. Co-pyrolysis of SS with basaltic materials increased Ca, Mg, P, and micronutrient contents. Contrasting responses among elemental, thermochemical, and persistence-based indices showed that no single approach adequately evaluates C stability in mineral-enriched biochars. These findings support the potential use of mineral-enriched biochars as soil amendments and show that C stability requires integrated evaluation.

## Graphical abstract

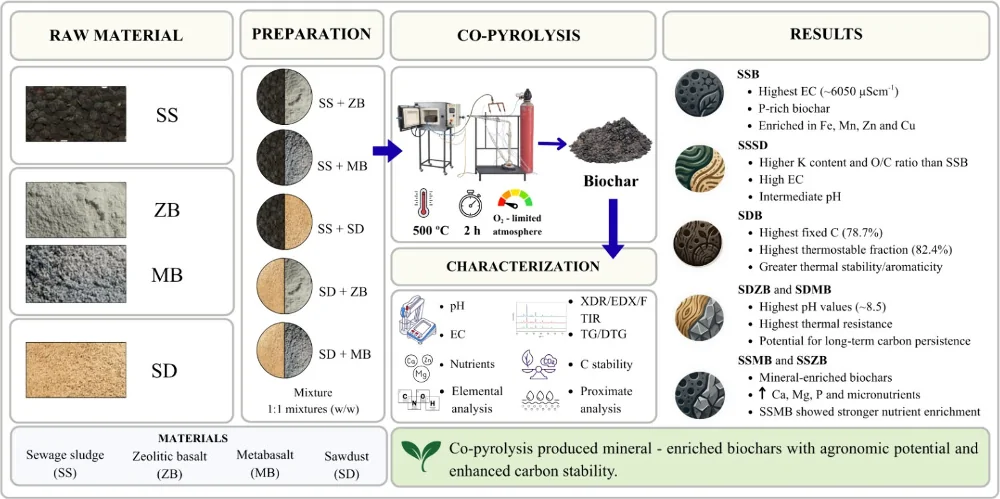

## 1 Introduction

The agricultural valorization of organic and mineral residues has emerged as a promising approach to advance the circular economy, recover nutrients, and reduce environmental impacts.^1^ Among residues with high potential are sewage sludge (SS), sawdust (SD), and basaltic materials from mining. However, their direct use can create agronomic and environmental challenges, requiring strategies that promote stabilization, safety, and value addition, particularly for soil amendments and long-term carbon (C) sequestration.

Sewage sludge is rich in organic matter and macro- and micronutrients, making it useful as a soil amendment.^2^ However, it may contain pathogens, heavy metals, and potentially toxic organic pollutants, requiring treatment before agricultural use.^3,4^ In the Federal District of Brazil, with about 3 million residents, roughly 400 tons of SS are produced daily.^5^ This SS mainly originates from domestic sources and contains high levels of organic matter and nutrients but has low potassium (K) content, along with heavy metals.^6^ Sawdust (SD), a lignocellulosic byproduct of wood processing, contains high amounts of C and volatile matter.^7,8^ Pyrolysis can stabilize these organic residues by removing volatile compounds and pathogens and forming more resistant aromatic C structures.^6,9^ The resulting biochar may contribute to long-term C persistence, although its properties depend on the feedstock and pyrolysis conditions.^10–14^ Basalts and their metamorphic counterparts are notable and widely used as soil remineralizers because they supply calcium (Ca), Mg, K, and silicon (Si) through gradual weathering.^15–17^ Beyond their agricultural use, their Ca-, Mg-, and Fe-bearing minerals may also favor stable carbonate formation and long-term CO_2_ storage.^18–20^ Metabasalts (MB) contain plagioclase, clinopyroxene, chlorite, and biotite.^21–23^ They are rich in SiO_2_, Al_2_O_3_, Fe_2_O_3_, CaO, MgO, and K_2_O, with CaO + MgO + K_2_O ≥ 9% and K_2_O ≥ 1%, meeting Brazilian standards for remineralizers.^24^ Their reactive silicates may promote gradual nutrient release and contribute to CO_2_ removal via mineral weathering and secondary carbonate formation, supporting long-term C immobilization.^25,26^

The simultaneous thermal conversion of SS or SD and mineral materials, under oxygen-limited conditions, known as copyrolysis, may improve biochar functionality by fostering synergistic interactions between organic and mineral components. Combining SS with lignocellulosic biomass can mitigate limitations of sludge-derived biochar, such as low C content and a poorly developed pore structure, while also aiding in the dilution and stabilization of metals originally present in the sludge. Conversely, adding mineral-rich materials may increase the ash content in sawdust-derived biochars, potentially enhancing their agronomic performance. Furthermore, incorporating basaltic residues can add value to mining byproducts and support their environmentally responsible reuse, contributing to the development of mineral-enriched biochars produced via copyrolysis with mineral additives that exhibit increased stability and resource recovery potential. Consistent with this perspective, studies summarized by Han et al.^27^ indicate that copyrolysis of SS can enhance key agronomic properties of sludge-derived biochar, including higher pH, improved nutrient retention (P, K, Ca, and Mg), and greater C stability compared with sludge pyrolyzed alone. For example, Wang et al.^28^ showed that copyrolysis with cotton stalks increased biochar alkalinity, cation exchange capacity (CEC), nutrient availability, surface area, and aromaticity. In addition, copyrolysis with mineral additives has demonstrated beneficial effects; Xiao et al.^29^ utilized SS and phosphate tailings to produce biochars capable of inhibiting harmful elements while enriching plant-available nutrients, and Guo et al.^30^ emphasized that copyrolysis of SS with K- and Ca-rich biomass ash improved nutrient bioavailability and fertilizer potential. Overall, these findings suggest that integrating mineral-rich residues during copyrolysis can enhance the agronomic performance of SS-derived biochar while increasing its environmental safety and long-term C stability, supporting its application in sustainable soil management.

Our previous research examined the pyrolysis and copyrolysis of SS and basaltic zeolite (ZB), demonstrating that mineral inclusion affects C stability, elemental composition, and crystalline phases.^31^ However, it remains unclear whether these effects are consistent across contrasting organic feedstocks and basaltic materials and how mineral dilution and silicate-rich phases influence the interpretation of conventional C-stability indices. We hypothesized that copyrolysis of SS, SD, and basaltic materials would modify the physicochemical, mineralogical, and C-stability properties of the resulting biochars through interactions between the organic and inorganic fractions during pyrolysis. This study compared SS- and SD-derived feedstocks copyrolyzed with ZB and MB and assessed their structural, chemical, thermal, mineralogical, soil amendment, and C-stability properties. The novelty of this study lies in the integrated comparison of two contrasting organic feedstocks and two basaltic materials and in evaluating how mineral incorporation affects different C-stability indicators.

## 2 Materials and Methods

### 2.1 Preparation of Feedstocks and Biochars Production

Four common organic and mineral materials sourced in Brazil—sewage sludge (SS), sawdust (SD), zeolitic basalt (ZB), and metabasalt (MB)—were selected for this study. For descriptions of their origin and feedstock preparation, see the Supporting Information. The feedstocks were pyrolyzed individually, whereas SS + SD, SS + ZB, SS + MB, SD + ZB, and SD + MB were prepared at a 1:1 (w/w) ratio and homogenized by vigorous stirring. The 1:1 (w/w) ratio was selected from preliminary trials to balance the contributions of both feedstocks, consistent with previous copyrolysis studies that used the same proportion.^31,32^ All materials were pyrolyzed in separate batches using a laboratory-scale fixed-bed reactor installed in an electric furnace. Before each batch, the reactor was purged with a continuous N_2_ flow of 1–2 L min^–1^, which was maintained throughout the process to ensure an inert atmosphere. Volatile products were directed through a water-cooled condensation system, where the condensable fraction was collected as bio-oil and noncondensable gases were vented to the atmosphere through the terminal gas outlet. All batches were heated to 500 °C at 10 °C min^–1^ and held at that temperature for 2 h. After pyrolysis, the system was allowed to cool to room temperature, and the biochar was then recovered, weighed, and stored in sealed containers. A schematic of the pyrolysis system is shown in Figure S1. For clarity, biochars derived from individual feedstocks were designated SSB, SDB, PZB, and PMB, corresponding to SS, SD, ZB, and MB, respectively. Co-pyrolyzed biochars were designated SSSD, SSZB, SSMB, SDZB, and SDMB.

The yields of biochar or bio-oil were determined using the following eq 1:

1 $$Yield\left(\%\right)=\left(\frac{W_{1}}{W_{0}}\right)\times 100$$

where *W*_1_ is the mass of biochar or bio-oil and *W*_0_ is the mass of the feedstock. The gas yield was obtained by subtracting the yields of biochar and bio-oil from 100%.

### 2.2 Electrical Conductivity, pH, and Nutrient Levels

The pH and electrical conductivity (EC) of the samples were measured using the electrometric method.^33^ Measurements were performed in deionized water at a 1:10 (w/v) ratio after shaking in an orbital shaker for 1 h. Macronutrients were extracted using nitroperchloric digestion, with P quantified by the molybdovanadophosphoric acid colorimetric method, K by flame photometry, and Ca, Mg, and S by inductively coupled plasma optical emission spectrometry (ICP-OES; ICPE-9000, Shimadzu, Japan).^34^ For total K analysis, selected samples were digested with hydrofluoric and perchloric acids following the Brazilian Ministry of Agriculture and Livestock protocol.^35^ Elemental concentrations were expressed as percentages (g kg^–1^) for P, Ca, Mg, C, K, and Fe, whereas Cu, Mn, and Zn were reported in mg kg^–1^. Quality assurance and quality control included reagent blanks, calibration with analytical standards, matrix spikes, and triplicate analyses. Calibration and blank responses were verified for each batch, and precision and accuracy were assessed using replicate agreement and spike recovery.

### 2.3 Proximate and Ultimate Analyses

Proximate analyses of feedstocks, biochars from each feedstock, and copyrolyzed biochars were conducted to determine moisture, volatile matter (VM), ash content, and fixed carbon (FC), following ASTM D1762-84,^36^ with all measurements performed in triplicate. The ultimate analysis was also conducted to determine the contents of C, hydrogen (H), and N using an automatic elemental analyzer Model EuroEA3000 CHNS-O (EuroVector S.p.An Italy), equipped with a thermal conductivity detector. The O content was calculated as the difference between C, H, N, and ash contents, given the material’s low sulfur content. Because O was estimated by difference, O/C ratios in mineral-enriched biochars may partially reflect contributions from inorganic oxides and silicates. The atomic ratios of O/C, H/C, and (N+O)/C were calculated from elemental contents to evaluate aromaticity, polarity, and potential persistence.

### 2.4 Structural, Mineralogical, and Thermal Analysis of Feedstocks and Biochars

The feedstock and biochar samples were previously dried in an oven at 105 °C until reaching a constant weight, then ground and sieved (<50 μm) to ensure uniformity before conducting structural, mineralogical, chemical, and thermal analyses. Elemental composition was determined by energy-dispersive X-ray fluorescence spectroscopy (XRF/EDX) using Shimadzu spectrometer (model EDX-720HS). Mineral phases were identified by X-ray diffraction (XRD) with a Rigaku diffractometer (model Ultima IV) using monochromatic Cu Kα radiation (40 kV/25 mA), scanned from 10° to 70° (2θ). Surface functional groups were analyzed via Fourier transform infrared spectroscopy (FTIR) using a Varian spectrometer (model FTIR 640), operating from 4000 to 400 cm^–1^, equipped with a DLaTGS detector. Thermal stability and decomposition behavior were assessed using thermogravimetric analysis (TGA) on a Shimadzu analyzer (model DTG-60H), allowing simultaneous TG and DTA measurements. TG and DTG curves were recorded to evaluate the thermal stability of the materials.

### 2.5 Thermostable Fraction

The thermally stable fraction (TSF) was determined by the ratio between FC and the sum of VM and FC, according to eq 2:

2 $$TSF=\left(\frac{FC}{VM+FC}\right)\times 100$$

The FC content in one year (y) for biochar, based on feedstock and application (CCy,t), was calculated as the product of its mass, a permanence factor, and the value obtained for organic C (OC) from the elemental analysis^37,38^ (eq 3):

3 $$CCy,t=M_{y,t}\times F_{cp}\times PR_{de}$$

where M_y,t_ is the mass of biochar applied and used in one year (y), assuming 1 ton; PR_de_ is the permanence factor, applied according to the H/Corg ratio. For H/C < 0.4, the factor is 0.74; for H/C > 0.4, the applied factor is 0.56; and F_cp_ is the OC, obtained through elemental analysis.^39^

### 2.6 Potential of Biochar to Sequester C

The potential for C (CS) sequestration is calculated by eq 4:

4 $$CS=\frac{M_{fd}\times Y_{bc}\times C_{bc}\times R_{50}}{M_{fd}\times FC_{fd}}$$

where M_fd_ is the mass of the feedstock; Y_bc_ is the yield of biochar; C_bc_ is the C content; R_50_ is the recalcitrance index of the biochar; FC_fd_ is the C content of the feedstock.

To determine the R_50_ index, thermogravimetric data obtained from TGA (Section 2.4) were used. The data were corrected for ash and moisture contents, and the R_50_ index was calculated using eq 5.^40^

5 $$R_{50,x}=\frac{T_{50,x}}{T_{50graphite}}\times 100$$

where R_50,x_ represents the thermal recalcitrance index of biochar, T_50,x_ is the temperature corresponding to 50% mass loss of the biochar during oxidation, and T_50graphite_ is the temperature at 50% mass loss of graphite.

### 2.7 Evaluation of the C Stability of Biochar

The methodology established by the International Biochar Initiative (IBI) was used to estimate the fraction of biochar C likely to persist in soil over 100 years, based on atomic H/C and O/C ratios from elemental analysis. These ratios serve as indicators of aromaticity and C stability in soil. According to IBI criteria, biochar stability is categorized into three groups based on the H/C atomic ratio: high stability (H/C ≤ 0.4; expected persistence >1000 years), medium stability (0.4 < H/C ≤ 0.7; 100–1000 years), and low stability (H/C > 0.7; < 100 years).^41,42^

### 2.8 Statistical Analyses

The Shapiro-Wilk test (α = 0.05) was used to assess the normality of the data, while homogeneity of variances was verified using Levene’s test. All statistical analyses were performed using three analytical replicates for each material. The data were subjected to analysis of variance (ANOVA), and the means were compared using Tukey’s test (*p* < 0.05) with RStudio.

## 3 Results and Discussion

### 3.1 Product Yields and Mass Balance

The biochars exhibited distinct yields of pyrolysis coproducts (Figure 1). Bio-oil yields ranged from 10% to 25% for SSB and SDMB, respectively. Biochar yields followed the order: SSMB > SSZB > SDMB > SDZB > SSB > SSSD > SDB. The higher biochar yields in SSMB and SSZB were associated with lower bio-oil and gas production.

**Figure 1:**
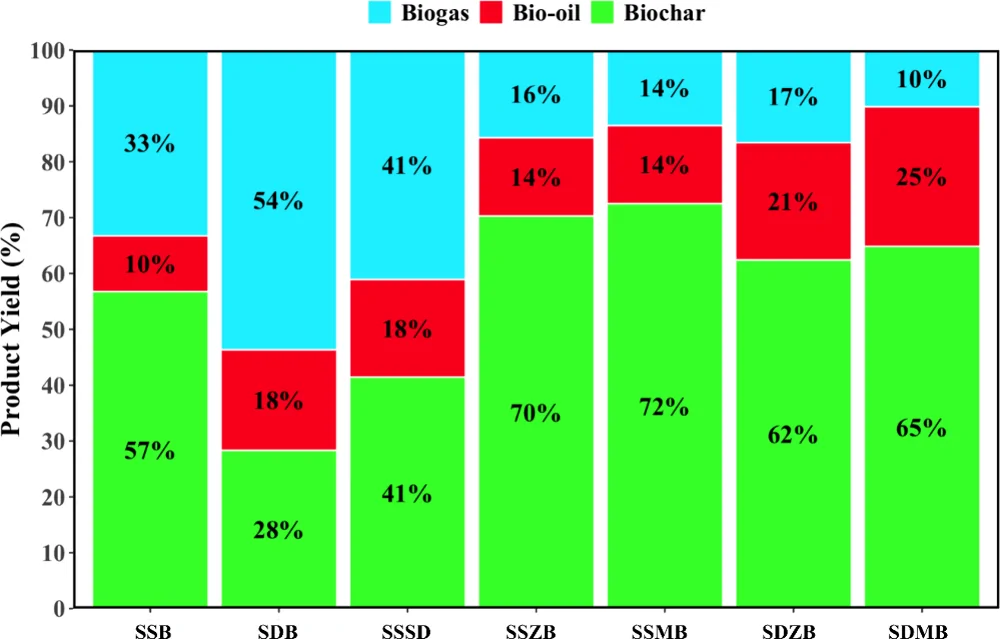
Biochar, biogas, and bio-oil yield from the materials. SSB, sewage sludge biochar; SDB, sawdust biochar; SSSD, copyrolyzed SS and SD biochar; SSZB, copyrolyzed SS and ZB biochar; SSMB, copyrolyzed SS and MB biochar; SDZB, copyrolyzed SD and ZB biochar; SDMB, copyrolyzed SD and MB biochar.

These differences reflect variations in the inorganic composition of the feedstocks, as indicated by their ash content. Sewage sludge had a high ash content (45.1%), whereas SD had a very low ash content (0.85%), indicating lower mineral content and a higher proportion of organic matter susceptible to volatilization during pyrolysis, which favors bio-oil and gas formation. In contrast, ZB and MB had ash contents of around 95% (Table S1), leading to higher solid-phase yields in the copyrolyzed biochars containing these materials. Because these rock powders primarily consist of thermally stable minerals, they experience minimal mass loss at 500 °C, releasing only residual moisture. As a result, the mineral fraction remains in the solid phase and is concentrated in the final biochar, thereby increasing the overall yield.^31^

### 3.2 Mineralogical/Structural Transformations — XRD, EDX, FTIR, and TGA

#### 3.2.1 X-ray Diffractograms and Energy-Dispersive X-ray

X-ray diffraction analysis (XRD) revealed significant differences among SS, SD, ZB, and MB, as well as among their respective pyrolyzed and copyrolyzed materials (Figure 2). These results indicate that mineralogical transformations at 500 °C were primarily governed by feedstock crystallinity. Sewage sludge (SS) exhibited a complex crystalline assemblage dominated by quartz (SiO_2_), identified by reflections at 2θ ≈ 20.8°, 26.6°, 39–40°, and 50°, with the strongest peak at 26.6°. This region also includes signals from aluminum phosphate (AlPO_4_), consistent with P precipitation through Al-based coagulation during wastewater treatment.^43^ Kaolinite (Al_2_Si_2_O_5_(OH)_4_) and gibbsite (Al­(OH)_3_) were readily identified in the low-angle range (∼12–22°), indicating the presence of hydroxylated aluminosilicates and Al hydroxides. After pyrolysis (SSB), quartz and aluminum phosphate remained prominent, while reflections from kaolinite and gibbsite were significantly reduced or no longer visible, indicating dehydroxylation and partial structural collapse of these minerals at 500 °C.^44^

**Figure 2:**
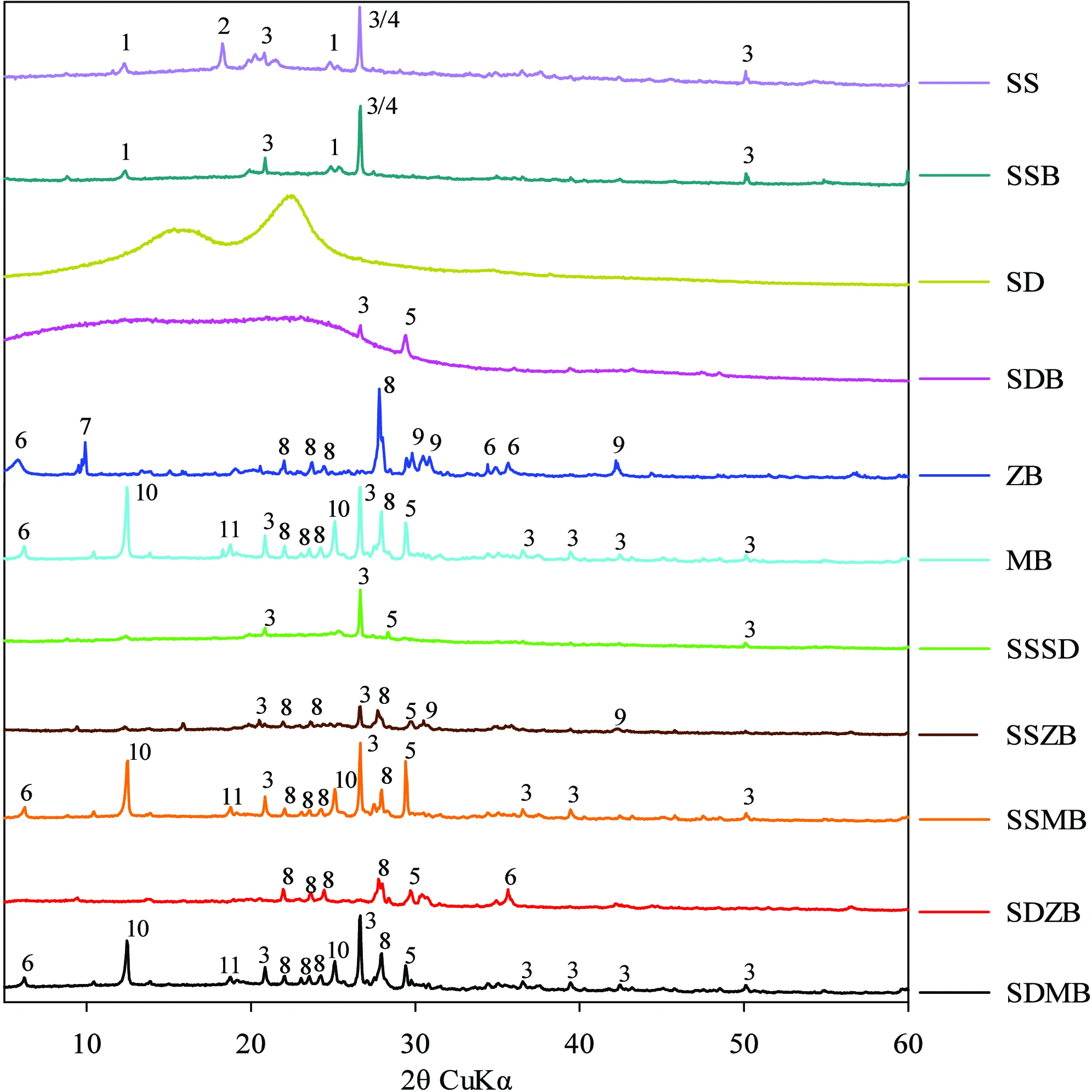
XRD spectra of the materials. SS, sewage sludge; SSB, sewage sludge biochar; SD, sawdust; SDB, sawdust biochar; ZB, zeolitic basalt; PZB, pyrolyzed zeolitic basalt; MB, metabasalt; PMB, pyrolyzed metabasalt; SSSD, copyrolyzed SS and SD biochar; SSZB, copyrolyzed SS and ZB biochar; SSMB, copyrolyzed SS and MB biochar; SDZB, copyrolyzed SD and ZB biochar; SDMB, copyrolyzed SD and MB biochar. 1: Kaolinite (Al_2_Si_2_O_5_(OH)_4_); 2: Gibbsite (Al­(OH)_3_); 3: Quartz (SiO_2_); 4: Aluminum phosphate (AlPO_4_); 5: Calcite (CaCO_3_); 6: Saponite (Ca_0_._25_(Mg,Fe)_3_((Si,Al)_4_O_10_)­(OH)_2_·nH_2_O); 7: Chabazite-Na ((Na,K,Ca)_2_Al_2_Si_4_O_12_·6H_2_O); 8: Labradorite ((Ca,Na)­(Al,Si)_4_O_8_); 9: Augite ((Ca,Na)­(Mg,Fe,Al,Ti)­(Si,Al)_2_O_6_); 10: Clinochlore ((Mg,Fe)_5_Al­(AlSi_3_O_10_)­(OH)_8_); 11: Muscovite (KAl_2_(AlSi_3_O_10_)­(OH)_2_).

Sawdust exhibited a largely amorphous structure, with a broad halo around 18–25° (2θ), typical of lignocellulosic biomass.^45^ After pyrolysis (SDB), the material remained mostly amorphous; however, calcite (CaCO_3_) was clearly detected, as evidenced by its characteristic reflection near 29.4° (2θ). The presence of calcite exclusively in SDB indicates that carbonate crystallization was enhanced during pyrolysis. This process is probably linked to in situ solid–gas reactions between CaO, formed by the breakdown of Ca-bearing phases, and CO_2_, released during the thermal degradation of organic matter, a mechanism commonly reported for biomass ashes.^46^ Small quartz reflections were also observed, indicating a common impurity in wood-based materials.^47^

Zeolitic basalt (ZB) had a well-defined crystalline structure, predominantly composed of saponite ((Ca,Na)_0_._3_(Mg,Fe)_3_(Si,Al)_4_O_10_(OH)_2_·nH_2_O), labradorite ((Ca,Na)­(Al,Si)_4_O_8_), chabazite-Na (NaAlSi_2_O_6_·H_2_O), and augite (Ca­(Mg,Fe)­Si_2_O_6_). Saponite was identified by its basal reflection near 5.8° (2θ), while labradorite was identified by its main reflection around 27.8° (2θ). Chabazite-Na showed a characteristic peak at approximately 9.3° (2θ), and augite exhibited a diagnostic reflection near 30.6° (2θ), consistent with plagioclase and clinopyroxene typical of basaltic materials.^48^ Similarly, MB was characterized by five main crystalline phases: quartz, calcite, labradorite, clinochlore ((Mg,Fe)_5_Al­(AlSi_3_O_10_)­(OH)_8_), and muscovite (KAl_2_(AlSi_3_O_10_)­(OH)_2_). Labradorite displayed its principal reflection at 27.8° (2θ), while calcite showed a diagnostic peak near ∼ 29.4° (2θ). Quartz was identified by its diffraction peak at 26.6° (2θ). Clinochlore presented a basal reflection at ∼ 12.5° (2θ), and muscovite at ∼ 18° (2θ), consistent with the presence of metamorphic phyllosilicates.^15^ These silicate and phyllosilicate phases remained structurally preserved after copyrolysis at 500 °C, with no evident peak suppression or displacement.

The copyrolyzed biochars exhibited mineralogical patterns reflecting both dilution effects and mineral–carbon interactions. In SSSD, quartz inherited from SS remained dominant, while calcite became detectable, indicating that incorporating SD promoted carbonate stabilization during copyrolysis. The absence of kaolinite and gibbsite confirms that their thermal destabilization occurs regardless of blending. In SSZB and SSMB, the diffractograms showed a clear superposition of SS-derived phases (quartz and aluminum phosphate) with basalt-derived silicates. In SSZB, the intensity of the quartz peak at 26.6° decreased, indicating dilution within a silicate-rich crystalline matrix. No new crystalline phases were observed, suggesting that copyrolysis did not induce significant recrystallization and instead preserved the original silicate structures. In SDZB and SDMB, the crystalline fraction was dominated by basalt-derived minerals (saponite, chabazite, labradorite, augite, clinochlore, and muscovite), superimposed on an elevated amorphous background from pyrolyzed SD. In SDZB, calcite was clearly detectable, indicating carbonate formation promoted by biomass-derived carbon and CO_2_ during pyrolysis. In contrast, in SDMB, calcite reflections were also observed but are partly inherited from the original MB, suggesting that carbonate presence in this blend results from both pre-existing mineral phases and thermally induced carbonation processes.

XRD results show that pyrolysis at 500 °C selectively alters hydroxylated sludge minerals while preserving quartz, Al–P phases, and the main silicate structures of basalt-derived materials. Variations in carbonate presence and peak intensities across treatments suggest differences in feedstock composition and blending (copyrolysis), with no evidence of extensive new crystalline phases.

According to the EDX results, SS- and SSB-derived materials had higher Fe, Al, and P contents than CH- and CHB-derived materials, reflecting the intrinsic mineral compositions of SS. Detailed EDX data and discussion are provided in the Supporting Information (Table S2).

#### 3.2.2 Characterization by Infrared Spectroscopy

FTIR spectra (Figure 3) reveal clear differences between feedstocks (SS and SD), their respective biochars (SSB and SDB), and the copyrolyzed biochars (SSSD, SSZB, SSMB, SDZB, and SDMB), emphasizing the combined effects of thermal decomposition and mineral addition. The broad band at 3400–3700 cm^–1^, attributed to – OH stretching vibrations, is strong in both feedstocks (SS and SD), indicating abundant hydroxyl groups associated with adsorbed water and phenolic functional groups. As these groups are gradually removed at high temperatures, this peak disappears in the biochars.^49^ After pyrolysis, this band decreases significantly in SSB and, especially in SDB, reflecting dehydration and condensation reactions at 500 °C.^50^ Among the mineral-enriched biochars, residual absorption in this region is mainly observed in SSMB and SDMB, while it is weak or nearly absent in SSZB and SDZB. This may indicate that hydroxyl-containing mineral phases are better preserved in the MB-containing biochars, whereas dehydration or structural changes in zeolitic phases likely reduce the – OH signal in ZB-derived materials. The weakening of this band confirms a reduction in oxygenated functional groups and increased structural condensation in the C matrix after pyrolysis.

**Figure 3:**
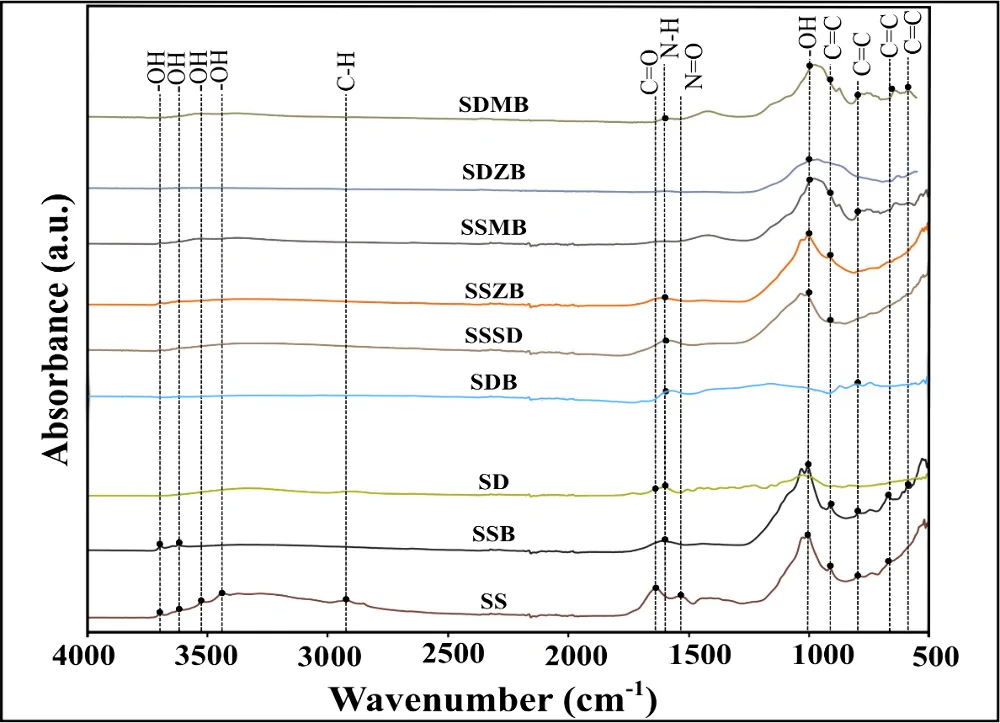
Fourier transform infrared (FTIR) spectra of the materials. SS, sewage sludge; SSB, sewage sludge biochar; SD, sawdust; SDB, sawdust biochar; SSSD, copyrolyzed SS and SD biochar; SSZB, copyrolyzed SS and ZB biochar; SSMB, copyrolyzed SS and MB biochar; SDZB, copyrolyzed SD and ZB biochar; SDMB, copyrolyzed SD and MB biochar.

The aliphatic C–H stretching bands at 2920 and 2851 cm^–1^ are prominent in SD and clearly visible in SS, confirming the presence of labile organic structures.^51^ These bands nearly disappear in SSB, SDB, and all copyrolyzed materials, indicating breakdown of aliphatic chains during pyrolysis at 500 °C. Signals in the fingerprint region (about 914, 750, 650, and 580 cm^–1^) became clearer in SDB and especially in SSB, indicating increased aromatic condensation after pyrolysis.^52^ Conversely, in mineral-enriched biochars (SSZB, SSMB, SDZB, and SDMB), the relative intensity of these bands is reduced, suggesting that inorganic phases dilute the carbonaceous matrix rather than inhibit aromatic structure formation. The band around 1600 cm^–1^ was more prominent in SS and in the biochars derived from SS, indicating the presence of amide bonds and/or aromatic ring stretching related to C=O and C=C vibrations.^31,53^ The band at 1540 cm^–1^ (N=O), clearly visible in SS, disappears in SSB and all copyrolyzed materials, consistent with N loss and transformation during heating.^53^ This matches the observed decrease in total N after pyrolysis, as will be seen in Table 2. A strong band near 1000 cm^–1^ appears in all samples. In SDB and SSB, this band mainly comes from the vibration of C–O or O–H functional groups.^54^ However, in mineral-enriched biochars (SSZB, SSMB, SDZB, and SDMB), this region likely overlaps with Si–O stretching vibrations from silicate minerals,^55^ which explains its continued presence and relative strength even after the organic functional groups diminish.

#### 3.2.3 Thermogravimetric Characterization

The thermogravimetric (TG/DTG) profiles show notable differences in mass-loss patterns and thermal stability among raw materials, single-feedstock biochars, and mineral-enriched biochars (Figure 4). The natural samples (SS and SD) experience the greatest weight losses, mainly between 200 and 500 °C. A mass reduction of 79.5% for SS and 91.9% for SD is observed up to 500 °C, primarily due to the release of volatile compounds. Sewage sludge exhibits a widespread and continuous degradation pattern, consistent with its diverse organic composition, which mainly includes proteins, polysaccharides, lipids, and other structurally varied organic compounds that decompose over a wider temperature range.^56^ In contrast, SD shows a more clearly defined DTG minimum around 350–450 °C, indicating a dominant decomposition event linked to the thermal degradation of hemicellulose and cellulose, which together make up about 86.62% of its composition.^57^ Both materials leave relatively low residual mass at 1000 °C, reflecting their high volatile content.

**Figure 4:**
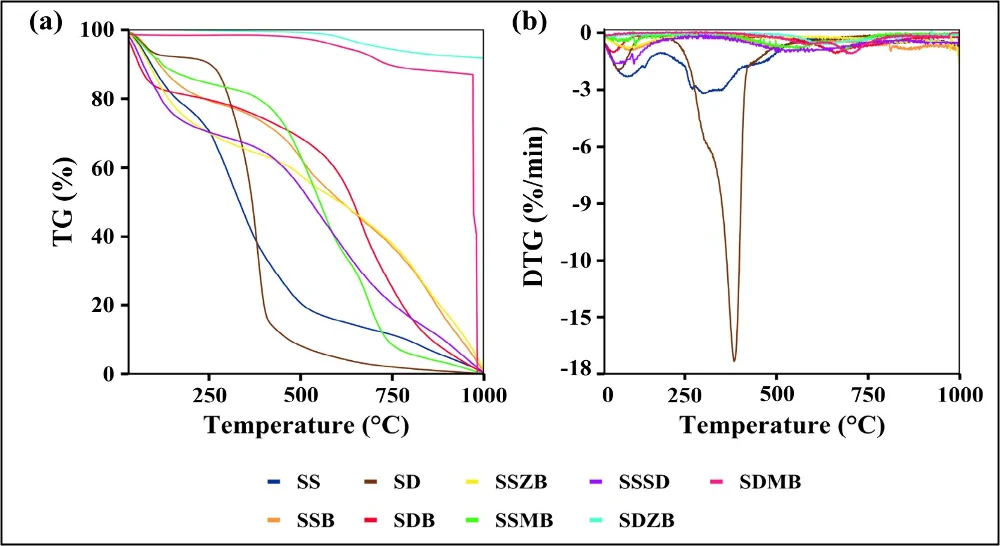
(a) Thermogravimetric (TG) curves and (b) derivative thermogravimetric (DTG) curves of the materials. SS, sewage sludge; SSB, sewage sludge biochar; SD, sawdust; SDB, sawdust biochar; SSSD, copyrolyzed SS and SD biochar; SSZB, copyrolyzed SS and ZB biochar; SSMB, copyrolyzed SS and MB biochar; SDZB, copyrolyzed SD and ZB biochar; SDMB, copyrolyzed SD and MB biochar.

Following pyrolysis, SSB and SDB display a substantially improved thermal resistance. The intense DTG peaks observed in the raw materials are largely suppressed, and the mass loss becomes more gradual, indicating the elimination of labile compounds and the formation of more condensed aromatic structures. SDB shows slightly greater stability at higher temperatures, suggesting a higher degree of C condensation, which may contribute to the long-term persistence of this biochar in soil.^58^

The mineral-enriched biochars (SSZB, SSMB, SDZB, and SDMB) retain the highest residual masses at 1000 °C and show flatter TG curves (Figure 4a) and reduced DTG signals (Figure 4b). This pattern indicates higher ash content from mineral components, which directly contributes to the higher residual mass. Additionally, phases containing Ca, Mg, and K can improve thermal resistance by forming refractory inorganic compounds during heating. According to Lane et al.,^59^ these elements promote the development of thermally stable mineral phases, reducing the volatilization of organic compounds and ultimately increasing the residual mass at high temperatures. Above 500 °C, all biochars undergo only slow and progressive mass loss, associated with the breakdown of more recalcitrant C domains and mineral transformations.

### 3.3 Proximate and Elemental Composition

#### 3.3.1 Proximate Analysis

The materials exhibited significant variation in proximate analysis parameters, the Ash/FC ratio, and the thermostable fraction (TSF) (*p* < 0.05), reflecting feedstock characteristics and the effects of pyrolysis and copyrolysis (Table 1). Moisture content (MC) was notably higher in the feedstocks, especially SD (7.15%), while pyrolyzed materials consistently showed lower values, particularly mineral-enriched biochars (≤2.21%). This reduction confirms the effectiveness of pyrolysis in removing physically bound water and improving material stability.

**Table 1:** Proximate Analysis, Ash/FC Ratio, and the Thermostable Fraction (TSF) of the Feedstocks, Single-Feedstock Biochars, and Co-Pyrolyzed Biochars^a^

Material	MC (%)	VM (%)	Ash (%)	FC (%)	TSF (%)	Ash/FC
SS	5.52 ± 0.21ab	47.9 ± 0.27b	45.1 ± 0.24f	7.01 ± 0.05f	12.8 ± 0.13h	6.43 ± 0.04c
SSB	1.37 ± 0.05c	18.0 ± 0.07c	68.4 ± 0.03c	13.6 ± 0.07e	43.0 ± 0.22e	5.02 ± 0.03c
SD	7.15 ± 0.08a	84.0 ± 0.29a	0.86 ± 0.13h	15.2 ± 0.16d	15.3 ± 0.18g	0.06 ± 0.01c
SDB	0.61 ± 0.03c	16.8 ± 0.53d	4.56 ± 0.10g	78.7 ± 0.55a	82.4 ± 0.56a	0.06 ± 0.00c
SSSD	4.95 ± 0.70b	18.9 ± 0.10c	51.8 ± 0.10e	29.3 ± 0.17b	60.8 ± 0.25c	1.77 ± 0.01c
SSZB	2.21 ± 0.00c	10.2 ± 0.00f	86.0 ± 0.00a	3.77 ± 0.00g	26.9 ± 0.00f	22.8 ± 0.00b
SSMB	1.93 ± 0.06c	13.0 ± 0.08e	85.3 ± 0.21a	1.73 ± 0.14h	11.7 ± 0.80h	50.0 ± 3.99a
SDZB	0.79 ± 0.79c	10.1 ± 0.09f	67.0 ± 0.00d	23.0 ± 0.00c	69.7 ± 0.00b	2.91 ± 0.00c
SDMB	1.58 ± 0.01c	12.4 ± 0.15e	72.5 ± 0.40b	15.1 ± 0.36d	55.0 ± 0.63d	4.80 ± 0.14c

a Means followed by the same letters in the column are not significantly different according to the Tukey test (*p* < 0.05); mean values ± standard error of the mean (*n* = 3). MC, moisture content; VM, volatile matter; FC, fixed carbon; TSF, thermostable fraction; SS, sewage sludge; SSB, sewage sludge biochar; SD, sawdust; SDB, sawdust biochar; SSSD, copyrolyzed SS and SD biochar; SSZB, copyrolyzed SS and ZB biochar; SSMB, copyrolyzed SS and MB biochar; SDZB, copyrolyzed SD and ZB biochar; SDMB, copyrolyzed SD and MB biochar.

Volatile matter (VM) was markedly higher in SD (84.0%) than in SS (47.9%) (*p* < 0.05), consistent with the lignocellulosic nature of SD. Pyrolysis significantly decreased VM in all treatments (*p* < 0.05), especially in SSB and SDB, indicating extensive devolatilization. Among copyrolyzed biochars, VM remained higher in SSSD than in the mineral-enriched biochars (SSZB, SSMB, SDZB, and SDMB), implying that mineral addition further reduced the relative proportion of volatile organic fractions.

The ash content varied significantly among treatments (*p* < 0.05) and showed an inverse trend relative to VM. SS already had a high ash content, as made evident by its derived biochar (SSB). The highest ash levels (*p* < 0.05) were found in SSZB and SSMB, consistent with their combination of SS and mineral materials (ZB or MB), both known for their high inherent ash contents. Conversely, SD and SDB had significantly lower ash contents (*p* < 0.05), aligning with the mostly organic nature of sawdust. In biochars derived from SS, especially those copyrolyzed with mineral additives, this dominance of inorganic constituents, although it may limit long-term C sequestration capacity, could be advantageous for correcting soil acidity and supplying specific mineral elements, depending on ash composition.^60^ Fixed carbon (FC) exhibited an opposite trend compared with ash. SDB had the highest FC (*p* < 0.05), indicating greater preservation of recalcitrant C structures. Adding SD to SS (SSSD) significantly increased FC compared with SSB, while the addition of mineral materials (SSZB and SSMB) decreased FC due to dilution by inorganic phases. Since FC is positively linked to aromatic and thermally stable C structures,^61^ these findings indicate that copyrolysis with SD enables the incorporation of stable C even into predominantly mineral materials (ZB and MB), thereby creating mineral-enriched biochars with enhanced C stabilization potential.

The thermostable fraction (TSF) also varied significantly (*p* < 0.05), with SDB showing the highest value. Sludge-derived materials had significantly lower TSF values, indicating reduced formation of condensed aromatic structures. In general, TSF trends closely aligned with FC behavior, reinforcing the role of lignocellulosic biomass in promoting the development of thermally stable C. Higher TSF values observed in sawdust-derived biochars (SDB and the copyrolyzed biochars containing SD) indicate a greater potential for long-term C persistence in soil, thereby increasing their contribution to long-term C sequestration and partially offsetting CO_2_ emissions when applied as soil amendments.^62^

The ternary diagram (Figure 5a), based on VM, FC, and ash content, illustrates the compositional redistribution promoted by pyrolysis and copyrolysis within the environmental and agronomic performance framework. The ternary diagram was interpreted using four zones—environmental potential, environmental restriction, agronomic potential, and agronomic restriction—defined by the relative proportions of VM, FC, and ash content, following Silva et al.^60^ The feedstocks occupy distinct zones: SD is in the agronomic potential zone, reflecting its high VM and low ash content, characteristics associated with short-term nutrient availability and soil conditioning potential. In contrast, SS is positioned in the agronomic restriction zone due to its elevated ash content and moderate VM, which may limit its agronomic suitability when applied as a nonpyrolyzed material.

**Figure 5:**
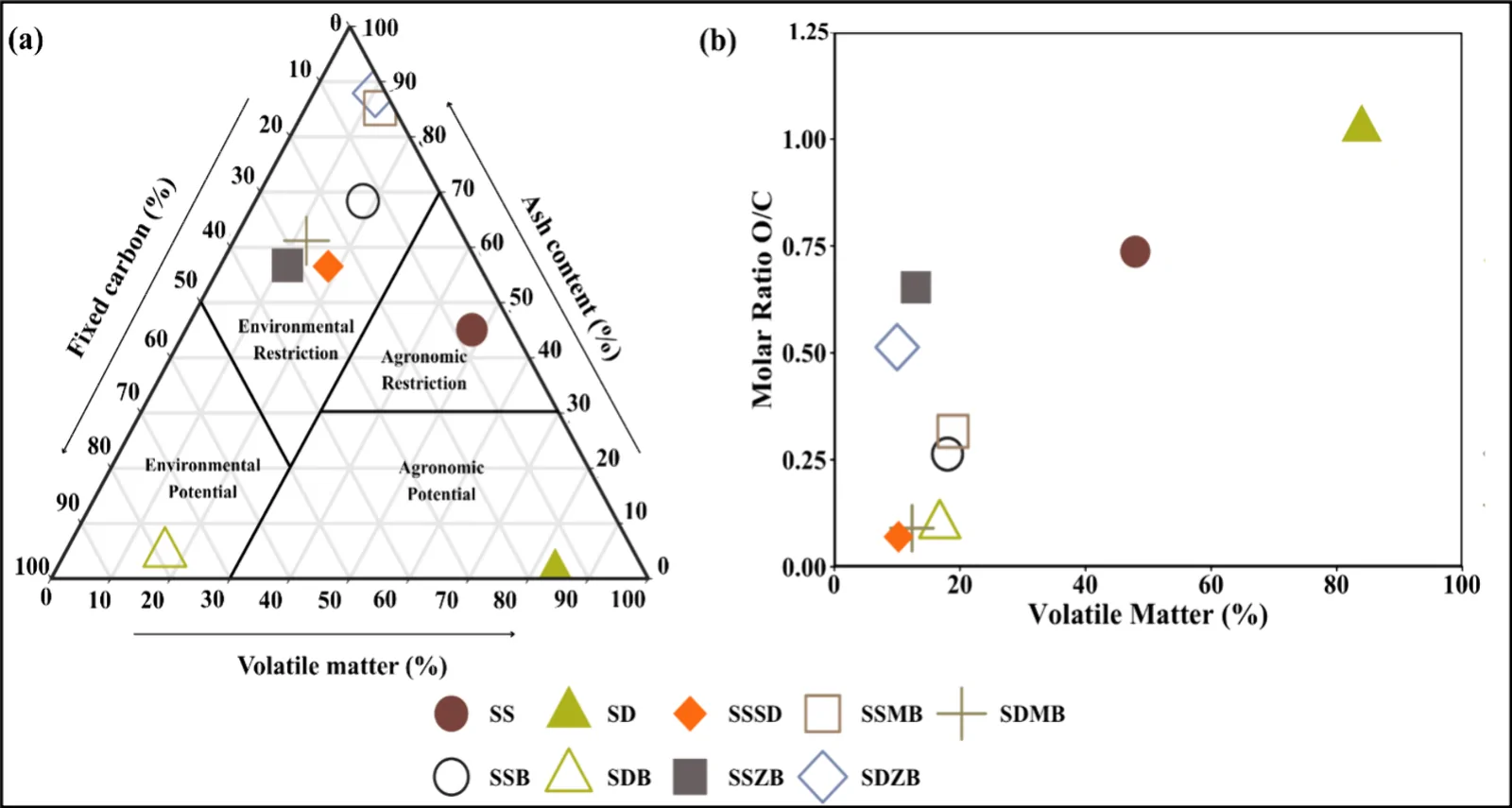
Ternary diagram (a) and relationship between volatile matter and O/C atomic ratio (b) of the materials. SS, sewage sludge; SSB, sewage sludge biochar; SD, sawdust; SDB, sawdust biochar; SSSD, copyrolyzed SS and SD biochar; SSZB, copyrolyzed SS and ZB biochar; SSMB, copyrolyzed SS and MB biochar; SDZB, copyrolyzed SD and ZB biochar; SDMB, copyrolyzed SD and MB biochar.

After pyrolysis, the materials shift toward regions associated with an increased stability. SDB clearly falls within the environmental potential zone, characterized by a high FC and reduced VM, indicating enhanced recalcitrance and greater potential for long-term C persistence. This displacement indicates a progressive aromatization during pyrolysis.

The remaining biochars, including SSB and the copyrolyzed materials (SSSD, SSZB, SSMB, SDZB, and SDMB), are predominantly located within the environmental restriction zone, characterized by high ash content (>70%) and FC below 50%.^60^ Although pyrolysis reduced VM, their elevated ash fractions and moderate FC values limit their classification within the environmental potential zone.

In mineral-enriched biochars, this positioning mainly reflects the strong contribution of mineral phases. The ash is rich in oxides (e.g., CaO, MgO, and K_2_O), as observed in the metabasalts (CaO + MgO + K_2_O ≈ 13.4%, with K_2_O above regulatory minimum requirements), and potentially toxic elements remain below the limits established by Brazilian legislation.^48^ Therefore, when mineral-enriched materials are involved, this classification within the environmental restriction zone does not necessarily imply environmental risk. Rather, it indicates inorganic enrichment and dilution of FC. Environmental safety must be interpreted considering mineral composition, application rate, and regulatory thresholds, since trace elements are substantially diluted under realistic field conditions, and some are classified as essential for plant growth. Thus, in mineral-enriched biochars, the ternary framework primarily captures the reduced, relatively recalcitrant C content, while these materials may still provide agronomic benefits through nutrient supply and liming capacity. The relationship between VM and O/C ratio (Figure 5b) supports this interpretation. Sawdust biochar combines low VM and low O/C, reinforcing its classification within the environmental potential zone. In contrast, mineral-enriched biochars show low VM but higher O/C ratios, indicating contributions from O-rich mineral phases rather than oxygenated OC.

#### 3.3.2 Ultimate Analysis

The elemental composition, atomic ratios, and polarity indices varied significantly among materials (*p* < 0.05), reflecting inherent feedstock characteristics and thermochemical changes induced by pyrolysis, especially in C concentration and the reduction of H-, O-, and N-bearing functional groups (Table 2). Carbon content clearly distinguishes lignocellulosic (SD) from sludge-derived materials (*p* < 0.05). Sawdust showed higher C (40.1%) than SS (23.4%), which is consistent with its predominantly organic composition.^32^ Pyrolysis significantly increased the C content of sawdust-derived biochar (SDB, 81.4%), indicating extensive carbonization and concentration of the carbonaceous fraction. This behavior results from thermal decomposition and structural rearrangement during pyrolysis, in which carbonaceous components are transformed into more stable, complex aromatic structures.^49^ Simultaneously, H- and O-containing functional groups (such as hydroxyl and carboxyl groups) are gradually volatilized, causing an increase in the C content and a corresponding decrease in H and O contents.^63^ Conversely, SSB had a slightly lower C (20.3%) than SS due to the high ash content in sludge, which probably dilutes OC after thermal treatment. Among copyrolyzed materials, SSSD (31.7%) displayed intermediate C levels, confirming that blending SD with SS promotes C enrichment. Mineral-enriched biochars produced via copyrolysis with mineral additives showed contrasting behavior: SSZB (10.6%) and SSMB (7.30%) had the lowest C contents due to strong mineral dilution, whereas SDZB (19.0%) and SDMB (23.5%) exhibited higher C contents, reflecting the higher contribution of SD-derived C despite mineral dilution.

**Table 2:** Elemental Composition of the Feedstocks, Single-Feedstock Biochars, and Co-Pyrolyzed Biochars^a^

Material	C (%)	H (%)	N (%)	O (%)	O/C	H/C	(N+O)/C
SS	23.4 ± 0.10d	4.58 ± 0.21a	4.02 ± 0.07a	23.0 ± 0.33b	0.74 ± 0.01ab	2.35 ± 0.10a	0.89 ± 0.01ab
SSB	20.3 ± 0.38d	1.42 ± 0.03c	2.73 ± 0.06b	7.18 ± 0.36cde	0.26 ± 0.02de	0.84 ± 0.02c	0.38 ± 0.02 cd
SD	40.1 ± 3.09b	5.18 ± 0.41a	0.27 ± 0.00e	53.6 ± 3.62a	1.02 ± 0.15a	1.55 ± 0.00b	1.03 ± 0.15a
SDB	81.4 ± 1.47a	2.94 ± 0.24b	0.70 ± 0.19d	10.4 ± 1.13cd	0.10 ± 0.01e	0.43 ± 0.04d	0.10 ± 0.01d
SSSD	31.7 ± 1.04c	1.39 ± 0.13cd	1.84 ± 0.05c	13.2 ± 1.20c	0.32 ± 0.04cde	0.53 ± 0.03d	0.37 ± 0.04 cd
SSZB	10.6 ± 0.36e	0.92 ± 0.07cd	1.46 ± 0.05c	0.98 ± 0.37e	0.07 ± 0.03e	1.04 ± 0.11c	0.19 ± 0.03 cd
SSMB	7.30 ± 0.76e	0.51 ± 0.05d	0.86 ± 0.10d	6.04 ± 0.74de	0.65 ± 0.16bc	0.83 ± 0.01c	0.76 ± 0.16ab
SDZB	19.0 ± 0.26d	0.76 ± 0.07cd	0.26 ± 0.05e	13.0 ± 0.21c	0.51 ± 0.01bcd	0.48 ± 0.05d	0.52 ± 0.02bc
SDMB	23.5 ± 0.16d	0.94 ± 0.04 cd	0.27 ± 0.04e	2.79 ± 0.49e	0.09 ± 0.02e	0.48 ± 0.02d	0.10 ± 0.02d

a Means followed by the same letters in the column are not significantly different according to the Tukey test (*p* < 0.05); mean values ± standard error of the mean (*n* = 3). SS, sewage sludge; SSB, sewage sludge biochar; SD, sawdust; SDB, sawdust biochar; SSSD, copyrolyzed SS and SD biochar; SSZB, copyrolyzed SS and ZB biochar; SSMB, copyrolyzed SS and MB biochar; SDZB, copyrolyzed SD and ZB biochar; SDMB, copyrolyzed SD and MB biochar.

Nitrogen (N) was significantly higher (*p* < 0.05) in SS compared to SD, probably because of the protein-rich nature of SS. Pyrolysis reduced the N content in all biochars, especially in SSB and SSSD, indicating volatilization of labile N compounds. This decrease mainly results from the thermal decomposition of N-containing structures, which releases gaseous species such as N_2_O, NO, and NO_2_ and converts residual N into more stable heterocyclic aromatic forms (e.g., pyridinic, pyrrolic, and quaternary N) during carbonization.^53,64^ Sawdust-derived biochars (SDBs) maintained low N levels, while mineral-enriched biochars generally showed further N dilution due to inorganic additives.

Hydrogen and O contents followed trends consistent with ongoing structural condensation during pyrolysis (*p* < 0.05). Sawdust initially had a similar H (5.18%) to that of SS (4.58%), but after pyrolysis, H decreased significantly in SDB (2.94%) and SSB (1.42%), indicating the formation of more condensed and aromatized C structures. A similar trend was observed for O in organic-rich materials, with SDB showing a sharp decrease, reflecting extensive devolatilization and the loss of oxygenated functional groups during pyrolysis.^32^ Conversely, the relatively high O content in mineral-enriched biochars (e.g., SSMB and SDZB) is primarily attributable to inorganic oxides^48^ rather than to organic oxygenated functional groups, highlighting the influence of mineral phases on elemental composition. Therefore, elemental analysis of mineral-enriched materials should be used with caution.

The atomic H/C ratios decreased significantly after pyrolysis (*p* < 0.05), with SDB and SD-derived biochars showing the lowest values (0.43–0.53), indicating highly condensed and stable C structures. In contrast, the feedstocks (SS and SD) had higher H/C ratios (2.35 and 1.55, respectively), reflecting less aromatic and more aliphatic structures. The O/C ratio followed the same general trend, with lower values in pyrolyzed materials (e.g., SDB and SDMB), consistent with advanced carbonization, while higher values in SS and SD reflect their noncarbonized nature and greater abundance of oxygenated groups. Intermediate values observed in mineral-amended biochars (SSMB and SDZB) are likely influenced by inorganic dilution. Notably, the very low O/C ratio in SSZB (0.07) is probably due to mineral dilution rather than enhanced C condensation.

### 3.4 Agronomic Properties — pH, EC, and Nutrients

#### 3.4.1 pH and EC

Biochars exhibited higher pH values than their respective precursors; however, a statistically significant difference (*p* < 0.05) was observed only for SD (Figure 6a). Although SS contains a substantial mineral fraction, its pH increased modestly after pyrolysis, a change that was not significant, possibly because of the greater buffering capacity of the inorganic constituents already present in the material. In contrast, SD, originally low in minerals, showed a more pronounced increase in pH, likely due to the degradation of acidic functional groups and the thermal transformation of inorganic constituents into mineral forms with basic character, such as carbonates, oxides, and hydroxides of alkali and alkaline-earth metals.^65^ In biochars obtained by copyrolysis, the effect on pH varied depending on the combination of raw materials. Copyrolysis of SS with SD resulted in intermediate pH values, with moderate elevation. However, the values obtained were similar (*p* > 0.05) to those observed for the individual biochars, showing no significant additive or interactive effect in the process.

**Figure 6:**
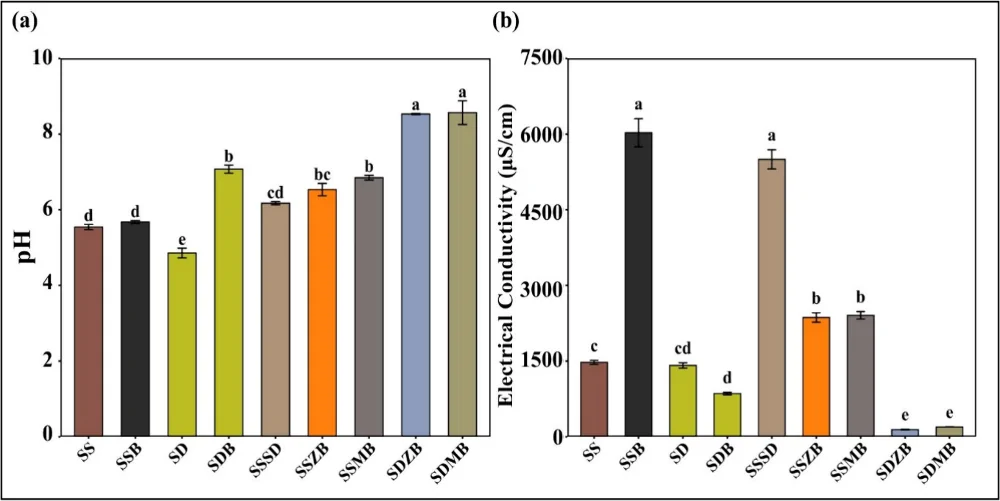
pH values (a) and electrical conductivity (EC) (b) of the materials measured in deionized water at a 1:10 solid:solution ratio after 1 h shaking. Different letters above the bars indicate significant differences among the biochars (Tukey test, *p* < 0.05). Error bars represent the standard error of the mean (*n* = 3). SS, sewage sludge; SSB, sewage sludge biochar; SD, sawdust; SDB, sawdust biochar; SSSD, copyrolyzed SS and SD biochar; SSZB, copyrolyzed SS and ZB biochar; SSMB, copyrolyzed SS and MB biochar; SDZB, copyrolyzed SD and ZB biochar; SDMB, copyrolyzed SD and MB biochar.

Copyrolysis with basaltic materials (ZB and MB) resulted in a more pronounced increase in pH, especially when combined with SD, whose treatments showed the highest values and differed significantly from the others (*p* < 0.05). Because pH was measured after a 1-h water extraction, the values reflect short-term alkalinity rather than the full neutralizing potential of slowly weathering basaltic minerals. These materials introduce silicates and oxides rich in Ca and Mg that, under pyrolysis conditions, can promote the formation or concentration of alkaline phases.^66^ In contrast, although SS also contains a relevant mineral fraction, its distinct mineralogical composition and the thermal behavior of its phases may limit the extent of the pH increases. The increase in pH is particularly relevant from an agronomic perspective, as higher values tend to correlate positively with CEC, thereby favoring cation retention and water dynamics in the soil.^67^ Additionally, the high alkalinity observed in copyrolyzed biochars (SDZB and SDMB) may intensify metal immobilization through precipitation, complexation, or specific adsorption reactions, expanding the potential of these materials for application in acidic or contaminated soils.^68,69^

Electrical conductivity (EC) varied significantly among the materials (*p* < 0.05) (Figure 6b). The SSB showed the highest value, statistically differing from the others, indicating a high concentration of soluble salts after pyrolysis. The copyrolysis of SSSD also resulted in a high EC, although lower than SSB and higher than the other biochars (*p* < 0.05). The biochars obtained by copyrolysis of SS with rocks (ZB or MB) showed intermediate values, higher than those of the feedstocks (SS, SD, ZB, and MB) and the biochars SDZB and SDMB, suggesting moderate release of soluble salts. Long-term application of biochars with elevated EC may influence soil physicochemical properties and structural stability, highlighting the importance of appropriate environmental monitoring and management to maintain soil health.^70^ Residues with EC values between 640 and 6050 μS cm^–1^ are generally considered suitable for agricultural use.^71^ Because EC reflects soluble salt content and potential salinity risk, mineral-enriched biochars may be useful in acidic soils, though application rates should account for the EC of SS-derived materials.

#### 3.4.2 Concentration of Macro- and Micronutrients

Pyrolysis promoted the redistribution and concentration of the mineral fraction in the individual biochars (Table 3). In the case of SS, conversion to SSB resulted in a relative increase in P_2_O_5_, Ca, and micronutrients such as Fe, Mn, Zn, and Cu, reflecting the degradation of the organic fraction and the retention of nonvolatile elements in the solid matrix. The P_2_O_5_ content in SSB (121 g kg^–1^) was substantially higher than that observed in SDB (2.00 g kg^–1^), demonstrating the high potential of SS-derived biochar as a P source. Unlike lignocellulosic biochars, which are generally poor in P, biochars produced from SS are naturally enriched with this nutrient and can increase the available P in the soil through the direct supply of endogenous P, with positive effects on crop productivity, as reported in the recent meta-analysis.^9^ However, K remained at low levels in SSB, indicating a limited contribution of this nutrient from the original material. Therefore, although SSB has high potential as a P source, its agricultural application may require K supplementation to adequately meet the nutritional demands of plants.^72^

**Table 3:** Organic Carbon (OC) and Macro- and Micronutrient Contents of the Feedstocks, Single-Feedstock Biochars, and Co-Pyrolyzed Biochars

Characteristic	SS	SSB	SD	SDB	ZB	PZB	MB	PMB	SSSD	SSZB	SSMB	SDZB	SDMB
OC^a^ (g kg^–1^)	283	149	555	291	23.0	12.0	20.0	19.0	198	48.0	59.0	104	118
K_2_O (g kg^–1^)	3.00	4.00	0.50	0.90	2.00	2.00	1.00	1.00	3.00	3.00	2.00	2.00	2.00
P_2_O_5_ (g kg^–1^)	66.0	121	1.00	2.00	3.00	6.00	3.00	5.00	84.0	40.0	40.0	3.00	3.00
Ca (g kg^–1^)	0.4.00	7.00	3.00	7.00	17.0	17.0	26.0	26.0	6.00	12.0	17.0	18.0	23.0
Mg (g kg^–1^)	2.00	3.00	1.00	0.2.00	8.00	8.00	25.0	25.0	3.00	7.00	15.0	9.00	21.0
S (g kg^–1^)	8.00	10.0	0.20	0.30	2.00	0.00	3.00	4.00	7.00	5.00	5.00	3.00	9.00
Fe (g kg^–1^)	19.1	30.1	2.59	1.26	16.1	18.1	61.0	63.1	30.5	25.1	59.1	19.5	44.2
Mn (mg kg^–1^)	62.0	95.0	31.0	64.0	249	298	2500	2595	86.0	247	1734	252	2016
Zn (mg kg^–1^)	434	715	14.0	21.0	43.0	45.0	495	483	492	259	583	63.3	329
Cu (mg kg^–1^)	126	198	9.00	5.00	80.0	65.0	129	123	134	131	153	91.7	108

a All concentrations are reported on a dry mass basis. SS, sewage sludge; SSB, sewage sludge biochar; SD, sawdust; SDB, sawdust biochar; ZB, zeolitic basalt; PZB, pyrolyzed zeolitic basalt; MB, metabasalt; PMB, pyrolyzed metabasalt; SSSD, copyrolyzed SS and SD biochar; SSZB, copyrolyzed SS and ZB biochar; SSMB, copyrolyzed SS and MB biochar; SDZB, copyrolyzed SD and ZB biochar; SDMB, copyrolyzed SD and MB biochar.

In the SD, the conversion to SDB was marked by a significant reduction in OC and increases in K and P_2_O_5_, as well as increases in the relative concentrations of Ca, Mg, and Mn after the loss of volatile compounds. The increase in nutrient concentration is attributed mainly to volatilization of organic compounds and the formation of more stable minerals, which are favored by the increase in pH from 4.85 to 7.07 (Figure 6a). The rise in pH promotes the precipitation and formation of less soluble mineral phases, increasing the retention of these elements in the organic-mineral matrix of the biochar.^73,74^ The reduction of OC from 555 g kg^–1^ in SD to 291 g kg^–1^ in SDB is associated with the progressive thermal degradation of the main lignocellulosic components. Between 300 and 400 °C, the decomposition of cellulose and hemicellulose predominantly occurs, generating vapors and both condensable and noncondensable gases.^75^ Considering that pyrolysis was conducted at 500 °C, there was also lignin degradation, as well as transformations and/or decomposition of some inorganic components.^76^

The copyrolyzed biochars exhibited chemical compositions that differed from those of materials obtained by individual pyrolysis, consistent with the combined contributions of the feedstocks during the thermal process. The biochar produced by copyrolysis of SS and SD showed increased levels of K and OC compared to SSB, suggesting a combined contribution of the mineral fraction of SS and the lignocellulosic fraction of SD to the final biochar composition. Similarly, the biochars obtained from copyrolysis of SS with ZB and SS with MB had higher levels of Ca and Mg compared to SSB, reflecting the incorporation of mineral fractions from rocks into the charred material. Basalt, for example, is recognized as a source of nutrients such as Ca, Mg, and micronutrients, which are gradually released through weathering processes from minerals like labradorite and diopside.^17,77^ Additionally, zeolites serve not only as a potential nutrient source but also, owing to their high CEC, favor the retention of elements released during SD pyrolysis.^78,79^ Specifically, the SSMB biochar showed high concentrations of P_2_O_5_, Ca, Mg, and micronutrients such as Fe, Mn, Zn, and Cu, suggesting its potential contribution to soil chemical improvement and plant nutrition because of the balance of macronutrients and micronutrients in the carbonized matrix. In the biochars produced by copyrolysis of SD with ZB or MB, increases in K and the alkaline earth cations (Ca and Mg) were also observed compared with SDB, showing that mineral addition substantially changed the chemical profile of the resulting biochar. While individual pyrolysis primarily increased the relative concentration of nonvolatile elements, copyrolysis produced distinct nutrient profiles, supporting the recycling of sludge-derived P and micronutrients, along with Ca and Mg from basaltic rocks, and the valorization of these residues. However, nutrient release and plant availability require validation under the soil conditions.

In addition to nutrient enrichment, the environmental implications of heavy metals (HMs) in SS-derived biochars should be considered. The HM concentrations in the original SS used in this study are presented in Table S1. Municipal SS is highly diverse, and its composition depends on factors such as the wastewater origin and treatment processes. Biosolids derived from nonindustrial sewage undergoing tertiary treatment typically exhibit lower heavy-metal levels and higher concentrations of essential nutrients, such as P and N.^80^ Furthermore, pyrolysis significantly mitigates the environmental impact of SS by altering HM speciation, reducing their bioavailability, and increasing oxidizable and residual HM fractions.^81–85^ Although SSB may retain higher total HM contents than raw sludge due to mass loss during carbonization, studies have shown that HM concentrations in SSB leachates are substantially lower,^86,87^ indicating enhanced immobilization of these elements after pyrolysis.

In this context, combining in situ and ex situ remediation strategies may be an important approach to prevent HM accumulation and ensure long-term soil health and safety in agricultural systems. Our previous field study showed that SSBs produced at 300 and 500 °C did not significantly increase the bioavailability of potentially harmful elements (Cd, Cr, Ni, and Pb) in soil over five years.^88^ Instead, the biochar exhibited a residual effect, enhancing the availability of plant micronutrients such as Cu, Mn, and Zn.

### 3.5 Carbon Stability Indices and Integrated Interpretation

#### 3.5.1 Indicators of C Stability and Sequestration Potential

The H/C and O/C atomic ratios are commonly used to assess biochar aromaticity and stability-related properties.^89^ Aromaticity in the biochar structure was evaluated using a van Krevelen diagram, in which decreases in H/C and O/C ratios are generally associated with increased aromatic condensation during pyrolysis. According to the International Biochar Initiative (IBI) and the European Biochar Certificate (EBC), H/C < 0.7 and O/C < 0.4 indicate well-carbonized and stable biochars.^90^ Under these criteria, SDB, SSSD, and SDMB fully met both thresholds (Figure 7). In particular, the low H/C ratios observed suggest enhanced aromaticity and structural stability of the C matrix, supporting the positive effect of SD incorporation during copyrolysis on C persistence. Additionally, the lowest (N+O)/C ratios were observed in SDB and SDMB (Table 2), indicating more hydrophobic and oxidation-resistant carbon structures.^91^

**Figure 7:**
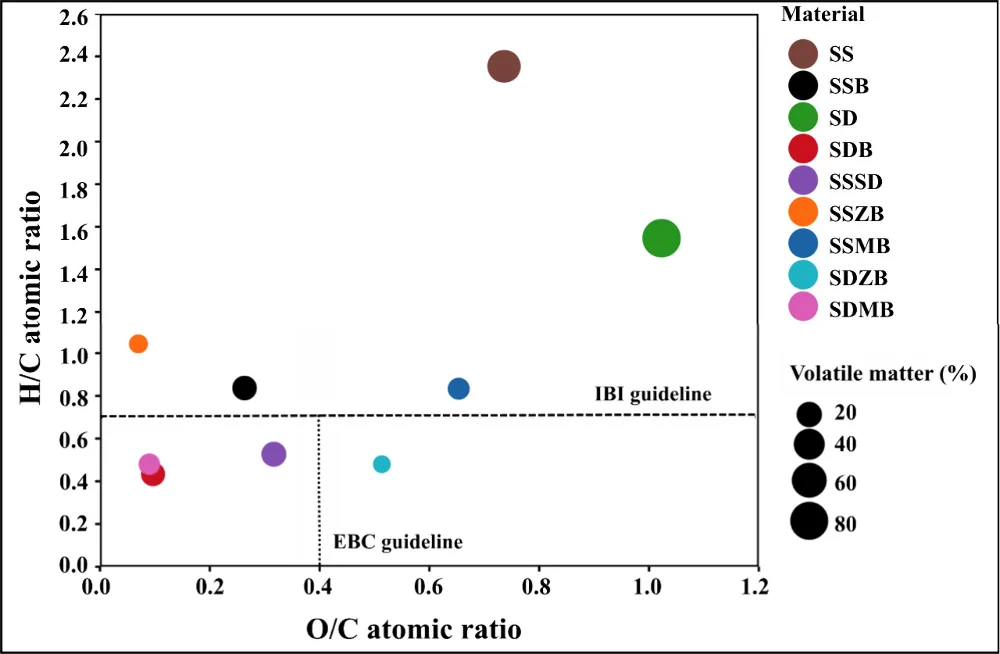
Van Krevelen diagram of the relationship between the atomic H/C and O/C ratios of the materials. Dotted line represents EBC criteria^90^ and dashed line represents IBI criteria^92^ to classify a charred material as biochar. SS, sewage sludge; SSB, sewage sludge biochar; SD, sawdust; SDB, sawdust biochar; SSSD, copyrolyzed SS and SD biochar; SSZB, copyrolyzed SS and ZB biochar; SSMB, copyrolyzed SS and MB biochar; SDZB, copyrolyzed SD and ZB biochar; SDMB, copyrolyzed SD and MB biochar.

Although O/C ratios are widely used as indicators of biochar stability and the degree of carbonization, caution is required when interpreting these values in mineral-enriched biochars. In the present study, biomass was blended with basalt/metabasalt at a 1:1 ratio prior to pyrolysis, resulting in materials with exceptionally high inorganic content. Consequently, part of the measured O is associated with mineral-derived phases, such as silicates and metal oxides, rather than exclusively with oxygen-containing functional groups in the carbonaceous matrix. Therefore, O/C ratios may not fully reflect the actual persistence potential of the C structure in these biochars and should be interpreted alongside complementary structural or oxidative stability analyses.^37,93^ Thus, the higher O-containing atomic ratios in mineral-enriched biochars likely reflect inorganic contributions, whereas the lower ratios observed for SSSD indicate that SD incorporation increased the structural stability of SS-derived C.

Remaining FC content in the soil in year y and time t (CCy,t) was used to assess the potential persistence of FC in soil (Figure 8a). Biochars derived from SD-containing feedstocks exhibited higher CCy,t values, indicating greater potential of FC to persist in soil over time.^37^ This behavior is associated with the higher FC and TSF observed for these materials (Table 1), suggesting the formation of more condensed C structures during pyrolysis, as commonly reported for lignocellulosic biochars.^94,95^ Conversely, biochars derived from the copyrolysis of SS and rocks showed comparatively lower CCy,t values, probably due to dilution of the carbonaceous fraction by the mineral matrix.

**Figure 8:**
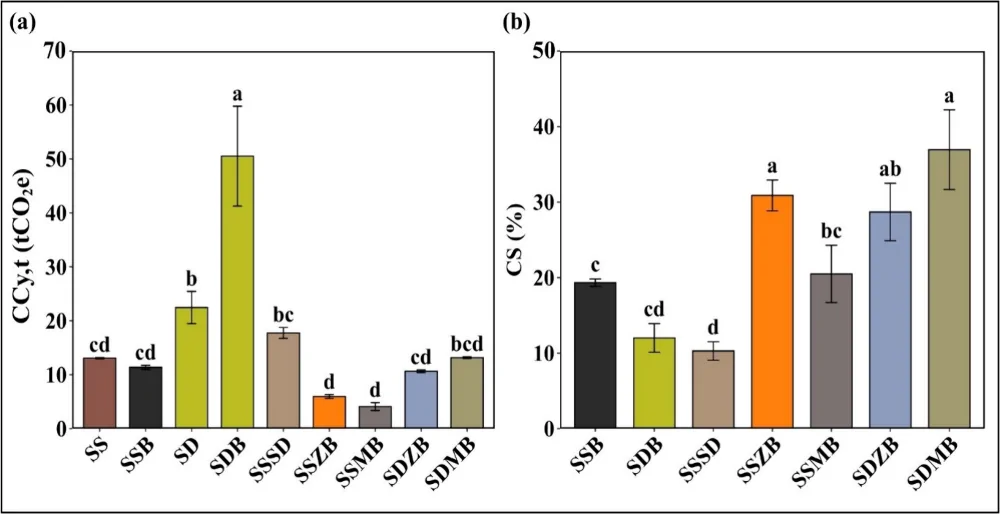
Remaining fixed carbon content in the soil in year y and time t (CCy,t) in (a) and C sequestration potential (CS) in (b) for the materials. Different letters indicate that the means were significantly different according to Tukey’s test (*p* < 0.05). Error bars represent the standard error of the mean (*n* = 3). SS, sewage sludge; SSB, sewage sludge biochar; SD, sawdust; SDB, sawdust biochar; SSSD, copyrolyzed SS and SD biochar; SSZB, copyrolyzed SS and ZB biochar; SSMB, copyrolyzed SS and MB biochar; SDZB, copyrolyzed SD and ZB biochar; SDMB, copyrolyzed SD and MB biochar.

Carbon sequestration potential (CS) showed a different pattern among the evaluated materials (Figure 8b). The highest CS values were obtained for SDMB, SDZB, and SSZB, which did not differ statistically. This pattern was consistent with the R_50_ values (Figure S2), which were substantially higher for these biochars than for the pristine SS and SD materials, confirming increased thermal recalcitrance after copyrolysis with rock powders. Thus, the comparatively high CS values of these materials were influenced not only by FC and OC contents but also by biochar yield and thermal resistance.^37^ However, the evaluation of CS in mineral-enriched biochars requires further study.

#### 3.5.2 Integrated Approaches for Evaluating Indicators of Biochar C Stability

Carbon stability did not respond uniformly across the evaluated indices, particularly for the mineral-enriched biochars. The contrasting classifications from elemental, thermochemical, and persistence-based approaches indicate that different indices capture distinct dimensions of biochar stability.^37,42^ The central finding is that feedstock composition and mineral content determine how each index responds; therefore, the stability of mineral-enriched biochars cannot be captured by a single metric (Figure 9). In general, biochars derived from SD-containing feedstocks, particularly SDB, exhibited higher stability according to most indices, including TSF, H/C, O/C, VM/FC, CCy, t, and CS. This behavior suggests that the lignocellulosic characteristics of SD favored the formation of more aromatic and recalcitrant C structures during pyrolysis, thereby contributing to greater C persistence in the resulting biochars.^94,96^

**Figure 9:**
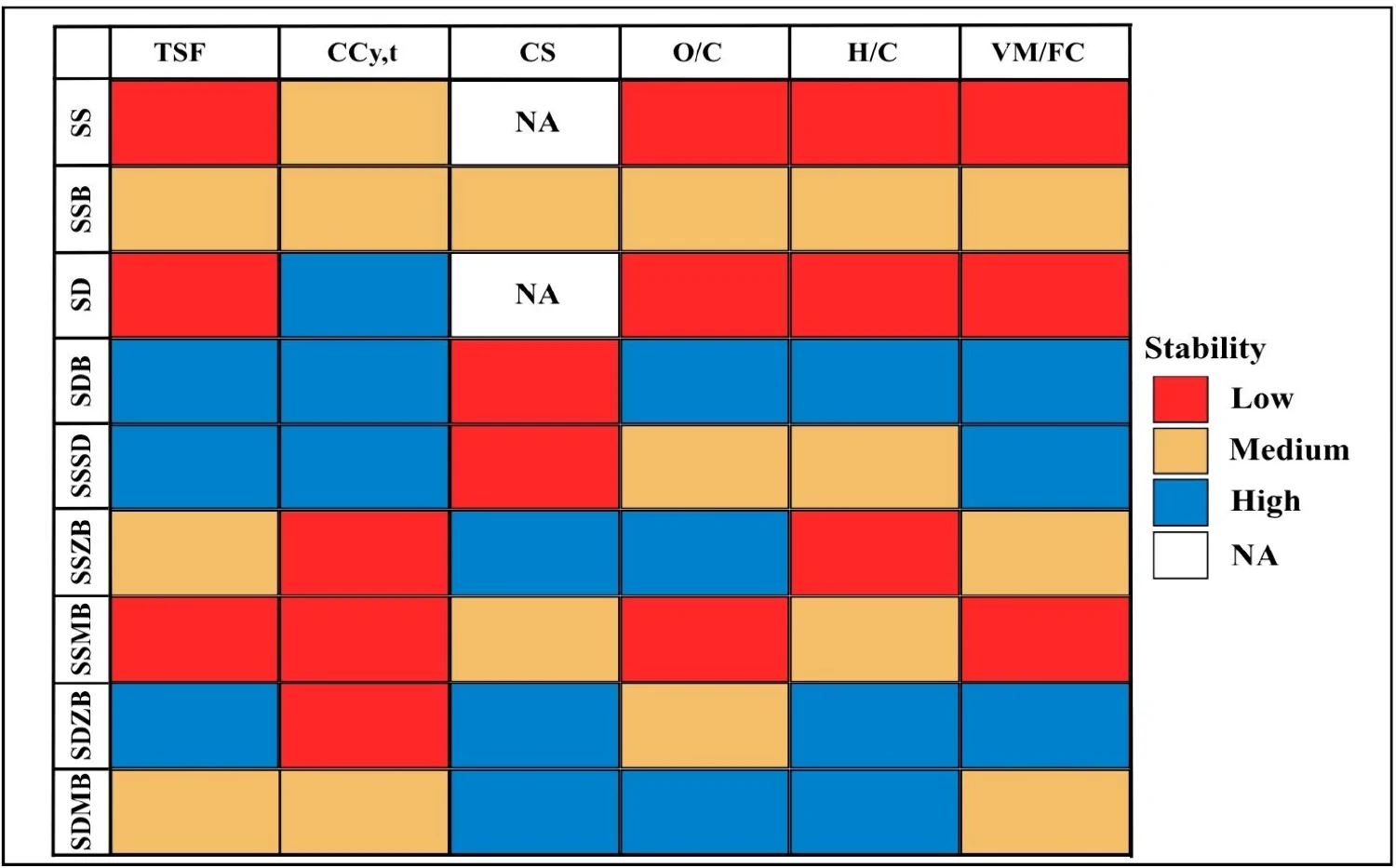
Carbon stability of the materials using different C stability methods. SS, sewage sludge; SSB, sewage sludge biochar; SD, sawdust; SDB, sawdust biochar; SSSD, copyrolyzed SS and SD biochar; SSZB, copyrolyzed SS and ZB biochar; SSMB, copyrolyzed SS and MB biochar; SDZB, copyrolyzed SD and ZB biochar; SDMB, copyrolyzed SD and MB biochar. TSF, thermostable fraction (%); CCy,t, remaining fixed carbon in the soil in year y and time t; CS, carbon sequestration potential; O/C, oxygen-to-carbon ratio; H/C, hydrogen-to-carbon ratio; and VM/FC, volatile matter/fixed carbon. The colors blue, orange, and red correspond to the highest, average, and lowest C stability, respectively.

The most significant discrepancies among the evaluated indices were observed in the mineral-enriched biochars. The sawdust-derived biochars enriched with basaltic zeolite or metabasalt (SDZB and SDMB) generally had high or intermediate stability according to TSF, CS, O/C, H/C, and VM/FC, indicating greater aromaticity, thermal resistance, and properties associated with long-term C persistence. Similarly, SSZB showed high stability according to CS and O/C, while TSF and VM/FC classified this material as having intermediate stability. In contrast, SSMB exhibited intermediate stability only for CS and H/C, whereas the remaining indices classified it as having low stability. These results demonstrate that the apparent stability of each material depends on the stability attribute measured by each index and on the relative contributions of the mineral and organic fractions during copyrolysis.

This divergence reflects the distinct principles of each approach. Indices such as H/C and O/C are primarily associated with aromatic condensation and elemental composition,^42^ whereas TSF is related to thermal recalcitrance.^37^ Conversely, CCy,t depends strongly on the FC content and is therefore directly influenced by ash dilution effects. Consequently, biochars with elevated mineral fractions may exhibit relatively low FC-based stability indices, despite high thermal resistance and aromaticity. Thus, disagreement among indices does not necessarily indicate inconsistency; rather, each index captures a distinct component of the C stability.

These contrasting responses highlight the limitations of applying conventional C stability indices to mineral-enriched biochars produced via copyrolysis with mineral additives. Because the inorganic fraction alters elemental ratios and dilutes FC and OC, isolated indices may underestimate the persistence of mineral-enriched biochars. The key takeaway is that C stability should be assessed by integrating elemental, thermochemical, and persistence-based indicators.

From a practical standpoint, the results indicate that feedstock selection can be used to tailor the functional potential of the resulting biochars. Sawdust-rich materials may be more suitable when long-term C persistence is prioritized. In contrast, SS–basalt combinations may be more relevant for nutrient recycling and soil acidity management because they combine SS-derived P and micronutrients with Ca-, Mg-, and silicate-rich mineral phases. However, their use should be adapted to soil conditions, since high ash content and alkalinity may benefit acidic soils, whereas elevated electrical conductivity may require appropriate application rates and monitoring. The simultaneous conversion of SS, SD, and mining residues also provides a pathway for waste valorization and the production of value-added soil amendments. Nevertheless, because C-stability indicators responded differently in mineral-rich materials, their contribution to C sequestration should be confirmed through soil incubation and field studies.

## 4 Conclusions

This study evaluated how copyrolysis of SS and SD with ZB and MB affects the physicochemical, mineralogical, thermal, nutrient-related, and C-stability properties of the resulting biochars. The copyrolysis increased biochar yield, ash content, and incorporated silicate-rich mineral phases, while also altering the concentrations of Ca, Mg, P, and micronutrients. Sawdust-derived biochars generally exhibited a higher fixed C content and stability according to conventional indices, whereas several mineral-enriched biochars showed high thermal recalcitrance despite their lower relative C contents. These contrasting responses demonstrate that mineral dilution and inorganic O-bearing phases can influence elemental ratios and carbon-based metrics, limiting the interpretation of any single C stability index. The main scientific contribution of this study is therefore the demonstration that C stability in mineral-enriched biochars should be evaluated through integrated elemental, thermal, mineralogical, and persistence-based approaches. Co-pyrolysis is a promising strategy for producing multifunctional biochars with distinct agronomic and C persistence-related properties, while also contributing to the valorization of SS, forestry residues, and mining byproducts. Further studies should be conducted to evaluate the stability indicators and C-sequestration potential under real soil conditions.

## Supplementary Material



## Notes

Details on feedstocks origin and preparation; physicochemical characteristics and pyrolysis yields of the feedstocks and pyrolyzed mineral materials (Table S1); and supplementary EDX results and elemental compositions of the feedstocks and biochars (Table S2); a schematic of the pyrolysis system (Figure S1); and the recalcitrance index (R_50_) of the feedstocks, single-feedstock biochars, and copyrolyzed biochars (Figure S2) (PDF)
