# Supplementary material for: Mineral-Enriched Biochars from Co-Pyrolysis of Sewage Sludge, Sawdust, and Basaltic Rocks: Soil Amendment Potential and Carbon Stability
Source: ACS Omega. 2026 Oct 19;11(43):67092–106. doi: 10.1021/acsomega.6c07681 (PMC13737925; doi:10.1021/acsomega.6c07681)
Supplement: Supplementary file 1 [file ao6c07681_si_001.pdf]

Supplementary Material for

Mineral-enriched biochars from co-pyrolysis of sewage sludge, sawdust, and basaltic rocks: soil amendment potential and carbon stability

Maíra Lopes D`Ávila[a], José Ferreira Lustosa Filho[a], Éder de Souza Martins[b], Giuliano Marchi[b], Cícero Célio de Figueiredo[a*]*

[a] Faculty of Agronomy and Veterinary Medicine, University of Brasília, 70910-970, Brasília, Federal District, Brazil

[b] Brazilian Agricultural Research Corporation, Embrapa Cerrados, 73310-970, Planaltina, Federal District, Brazil

Corresponding Authors

*Phone: +55-61-3107 7119. E-mail: filhoze04@hotmail.com.

*Phone: +55-61-3107-7564. E-mail: cicerocf@unb.br

- Number of pages: 7
- Number of figures: 2
- Number of tables: 2

Feedstock origin, preparation, and biochar production

The sewage sludge (SS) was obtained from the Samambaia Sewage Treatment Plant, operated by the Environmental Sanitation Company of the Federal District (CAESB) (15°52'21" S, 48°9'4" W). The plant uses a tertiary treatment system that, in addition to anaerobic decomposition, removes nutrients such as nitrogen (N) and phosphorus (P) from the effluent. These nutrients remain in the SS mass, which is then dried in covered areas with natural ventilation and masonry floors, preventing contact with the soil. In this process, nutrients like P and N are removed from the liquid effluent through coagulation with aluminum salts and remain in the final biomass of the SS.

The SS samples were air-dried (approximately 10% moisture content), ground in the TRO25 2Hp 3400 rpm Biv Tramontina organic grinder, and sieved through a 4 mm mesh. Next, the material was ground again in the TRE40MA electric forage grinder, with side outlet and a 3HP dual-voltage motor (Tramontina, model 79858416), and sieved through a 1 mm mesh. The ZB residue was collected from a quarry located in the Porto Franco region, Maranhão, Brazil (6°20’16″ S, 47°23’56″ W). The rock was crushed with a high-impact hammer and sieved through a 100-mesh screen (0.15 mm). The metabasalt (MB) rock powder samples were collected from a quarry in the Carajás region, Pará, Brazil (6°00’00″ S, 50°00’00″ W). The material was ground and classified by sieving, presenting a particle size between 0.15 and 0.30 mm and approximately 10% moisture content. The sawdust (SD), originating from the woodworking industry in northern Brazil, was ground and sieved through a 1 mm mesh.

Energy dispersive X-ray

According to the EDX results, SS- and SSB-derived materials had higher Fe, Al, and P contents than CH- and CHB-derived materials, reflecting the intrinsic mineral composition of SS (Table S2). In contrast, biochars produced with SD showed markedly

higher Ca contents, particularly in SD and SDB, whereas incorporating basaltic materials led to significant increases in Si, Mg, and Fe (Table S2). Among the mineral-enriched biochars, SSMB and SDMB exhibited the highest Fe concentrations, whereas SSZB and SDZB showed the highest Si contents, confirming the strong contribution of silicate-rich mineral phases from the rock materials. The EDX analysis also revealed the presence of several inorganic elements, including Al, Si, P, Ca, Fe, Ti, K, Mn, Zn, and Mg, indicating the formation of mineral-enriched biochars. In SD-derived materials, the reduction in Ca content after co-pyrolysis with basalt/metabasalt was accompanied by increases in Si, Fe, and Mg, suggesting partial mineral redistribution and the incorporation of rock-derived phases into the carbonaceous matrix. Similar mineral redistribution has been reported in mineral-amended biochars produced via co-pyrolysis with mineral additives, in which interactions between biomass ash and silicate- or metal-rich phases altered the inorganic composition, mineral speciation, and surface chemistry of the biochars [1]. Furthermore, the lower P contents observed in mineral-enriched biochars, especially those containing metabasalts, likely reflect dilution effects due to their high inorganic fraction. Because EDX provides semi-quantitative compositional data based on relative elemental proportions, the observed variations primarily reflect changes in mineral phase distribution rather than actual additions or losses of elements. These compositional changes indicate that co-pyrolysis with basaltic rocks alters the mineral profile of the biochars, potentially influencing their structural stability and agronomic behavior.

Table S1. Chemical and physical composition of sewage sludge (SS), sawdust (SD), non-pyrolyzed zeolitic basalt (ZB), non-pyrolyzed metabasalt (MB), pyrolyzed zeolitic basalt (PZB), and pyrolyzed metabasalt (PMB).

**Material**	**SS**	**SD**	**ZB**	**MB**	**PZB**	**PMB**
Biochar yield (%)	-	-	-		94.2	99,8
Bio-oil yield (%)	-	-	-		0	0
Biogas yield (%)	-	-	-		5.8	0,2
pH	5.50 ±	4.85 ± 0.15	6.70 ± 0.25	6.84 ± 0.21	7.40 ± 0.22	7.24 ± 0.05
EC (µS/cm)	1475 ± 38	1413 ± 3.16	187 ± 16.7	409 ± 1.05	288 ± 1.30	503 ± 1.43
Org. Mat. (%)	48.8	95.8	4.00	3.5	2.00	3.3
C/N ratio	3.40 ±	175 ± 15.5	9.91 ± 5.10	21.5 ± 12.5	11.8 ± 2.20	43.1 ± 11.2
Humidity (%)	5.50 ±	7.15 ± 0.08	1.97 ± 0.09	0.16 ± 0.01	0.20 ± 0.01	0.52 ± 0.33
Volatile matter (%)	47.2 ±	83.9 ± 0.29	6.05 ± 0.3	6.84 ± 0.04	2.70 ± 0.06	6.14 ± 0.32
Ash (%)	45.0 ±	0.86 ± 0.13	93.9 ± 0.3	93.2 ± 0.06	96.6 ± 0.02	92.8 ± 0.24
Fixed C (%)	7.00 ±	15.1 ± 0.16	0.03 ± 0.02	0.00 ± 0.00	0.58 ± 0.05	1.07 ± 0.08
Thermostable fraction	12.7 ±	15.3 ± 0.18	0.50 ± 0.30	0.00 ± 0.00	17.4 ± 1.60	14.9 ± 1.69
H/O ratio	2.00 ±	1.57 ± 0.20	0.00 ± 0.00	1.00 ± 0.05	0.81 ± 0.17	0.61 ± 0.04
FC/ash	6.40 ±	18.5 ± 2.96	2276 ± 438	0.00 ± 0.00	166 ± 8.60	0.01 ±
N (%)	3.30 ±	0.27 ± 0.00	0.02 ± 0.04	0.02 ± 0.02	0.04 ± 0.002	0.05 ± 0.05
Total C (%)	28.3	55.5	2.30	2.00	1.20	1.90
H (%)	5.20 ±	5.18 ± 0.41	0.14 ± 0.08	0.15 ± 0.07	0.15 ± 0.03	0.21 ± 0.01
H/C ratio	3.10 ± 0.1	1.55 ± 0.00	13.1 ± 3.42	13.1 ± 3.42	12.2 ± 3.27	1.79 ± 0.20
O (%)	26.4 ±	53.5 ± 3.62	5.90 ± 0.14	5.79 ± 0.14	3.02 ± 0.02	5.51 ± 0.32
O/C ratio	1.00 ±	1.02 ± 0.15	34.9 ± 4.79	34.9 ± 4.79	14.6 ± 1.00	2.99 ± 0.56
(O+N)/C	1.10 ±	1.03 ± 0.14	35.1 ± 4.80	35.1 ± 4.80	14.7 ± 0.99	3.01 ± 0.55
Cu, mg kg^{-1}	126	9.00	80.0	129.0	65.0	123
Mn, mg kg^{-1}	62.0	31.0	249	2500	298	2595
Zn, mg kg^{-1}	434	14.0	43.0	495	45.0	483
Cd, mg kg^{-1}	21					
Co, mg kg^{-1}	14					
Cr, mg kg^{-1}	86					
Ni, mg kg^{-1}	59					
Pb, mg kg^{-1}	142					

Table S2. Elemental composition (%) of feedstocks, single-feedstock biochars, and co-pyrolyzed biochars

Element	Material								
	**SS**	**SSB**	**SD**	**SDB**	**SSSD**	**SSZB**	**SSMB**	**SDZB**	**SDMB**
Fe	26.1	26.2	4.38	10.5	26.6	22.0	29.0	20.7	29.2
Al	26.0	24.2	6.04	4.67	28.4	24.0	15.8	14.7	13.0
P	15.6	12.5	-	-	15.5	11.0	3.63	-	-
Si	15.2	16.0	-	4.99	14.0	25.5	29.6	38.3	34.6
Ca	6.13	8.57	70.80	61.6	6.69	9.27	11.4	16.9	12.4
S	4.82	6.52	7.01	2.64	2.31	1.51	1.24	0.32	0.12
Ti	2.69	2.40	-	1.71	2.91	2.11	1.42	1.64	1.11
K	2.53	2.34	8.59	7.25	2.49	2.26	2.64	2.64	2.78
Zn	0.55	0.47	-	-	0.55	0.22	0.27	0.03	0.18
Cu	0.18	0.14	-	-	0.19	-	-	-	-
Mn	0.08	0.08	2.73	1.31	0.08	0.22	0.94	0.33	1.01
Cr	0.07	0.05	-	-	0.08	0.05	0.05	0.05	0.07
Sr	0.06	0.06	0.48	0.27	0.08	0.14	0.04	0.19	0.03
Mg	-	-	-	3.40	-	1.65	3.89	4.12	5.51
V	-	-	-	-	-	0.06	0.07	0.07	0.09
Sc	-	-	-	1.57	-	-	-	-	-
Zr	-	-	-	-	0.15	-	-	0.03	0.03

SS, sewage sludge; SSB, sewage sludge biochar; SD, sawdust; SDB, sawdust biochar; SSSD, co-pyrolyzed SS and SD biochar; SSZB, co-pyrolyzed SS and ZB biochar; SSMB, co-pyrolyzed SS and MB biochar; SDZB, co-pyrolyzed SD and ZB biochar; SDMB, co-pyrolyzed SD and MB biochar.

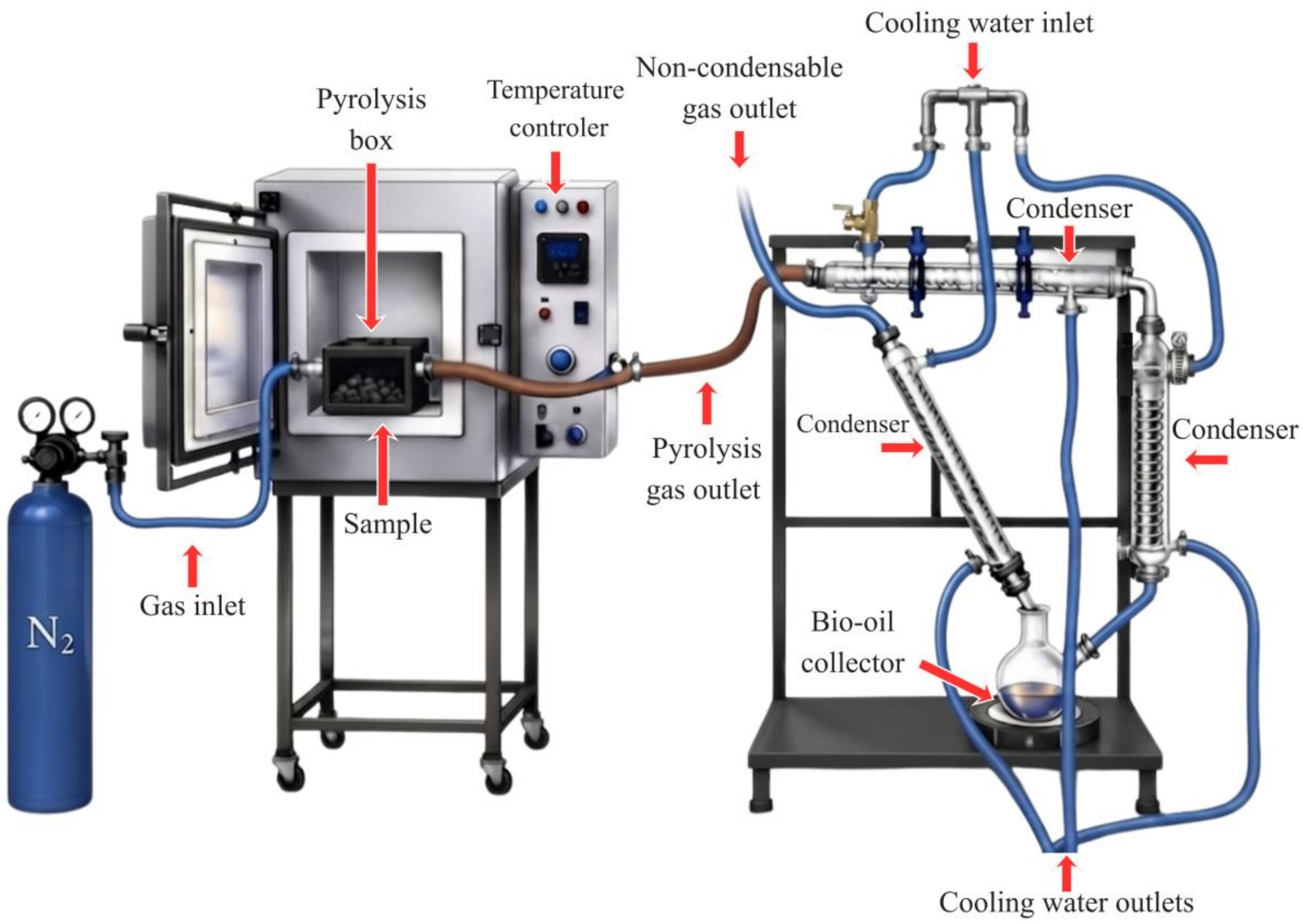

Figure S1. Schematic representation of the laboratory-scale pyrolysis system.

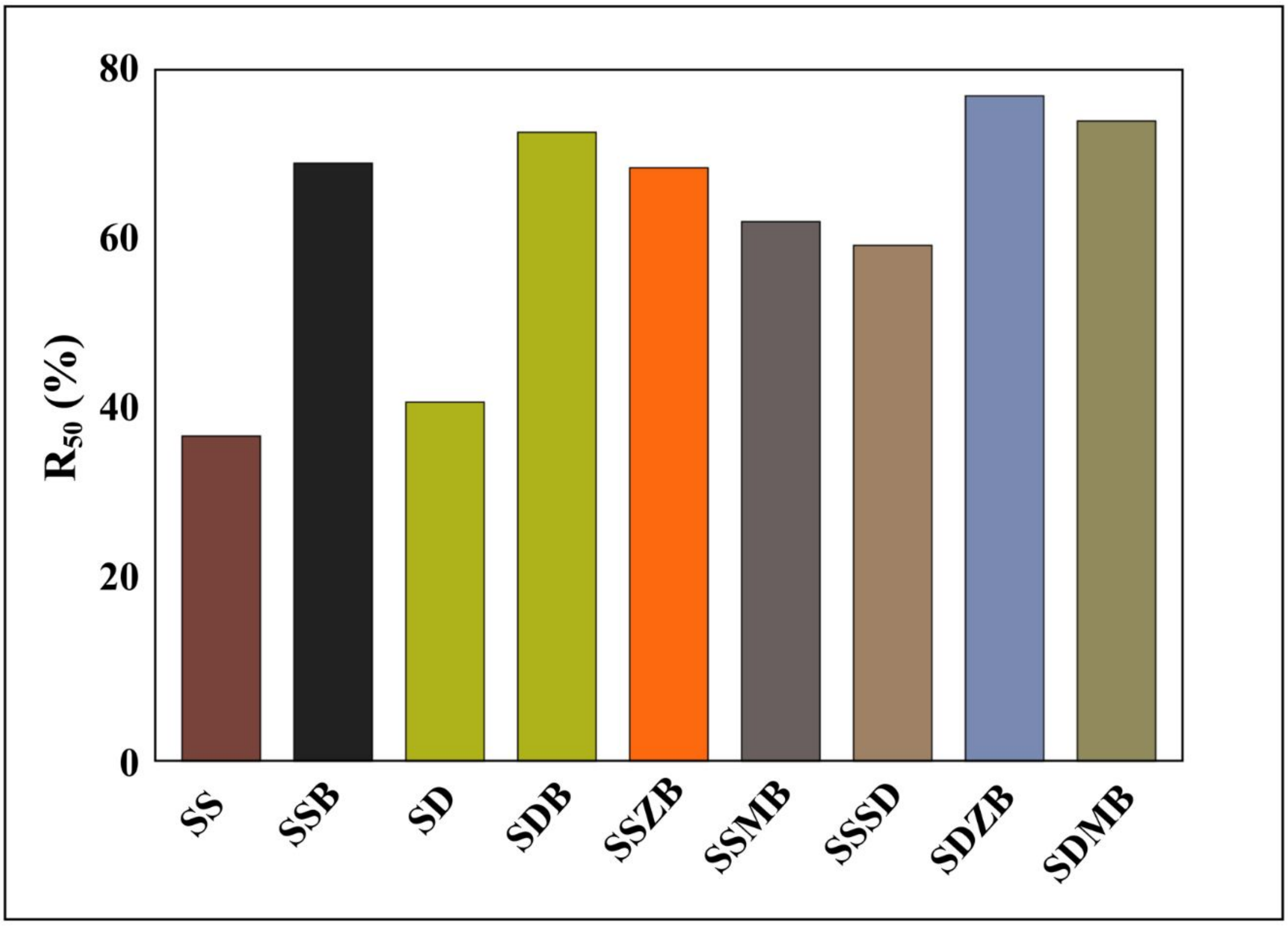

Figure S2. R_{50} index from feedstocks, single-feedstock biochars, and co-pyrolyzed biochars. SS, sewage sludge; SSB, sewage sludge biochar; SD, sawdust; SDB, sawdust biochar; SSSD, co-pyrolyzed SS and SD biochar; SSZB, co-pyrolyzed SS and ZB biochar; SSMB, co-pyrolyzed SS and MB biochar; SDZB, co-pyrolyzed SD and ZB biochar; SDMB, co-pyrolyzed SD and MB biochar.

References

(1) Wang, Z.; Xie, L.; Liu, K.; Wang, J.; Zhu, H.; Song, Q.; Shu, X. Co-Pyrolysis of Sewage Sludge and Cotton Stalks. *Waste Manag.* **2019**, *89*, 430–438. https://doi.org/10.1016/j.wasman.2019.04.033.
